# A chromosome-scale genome assembly of *Quercus gilva*: Insights into the evolution of *Quercus* section *Cyclobalanopsis* (Fagaceae)

**DOI:** 10.3389/fpls.2022.1012277

**Published:** 2022-09-23

**Authors:** Xia Zhou, Na Liu, Xiaolong Jiang, Zhikuang Qin, Taimoor Hassan Farooq, Fuliang Cao, He Li

**Affiliations:** ^1^ College of Forestry, Central South University of Forestry and Technology, Changsha, China; ^2^ Bangor College China, A Joint Unit of Bangor University and Central South University of Forestry and Technology, Changsha, China; ^3^ Co-Innovation Center for Sustainable Forestry in Southern China, Nanjing Forestry University, Nanjing, China

**Keywords:** *Quercus gilva*, PacBio sequencing, chromosome-scale genome assembly, phylogeny, evolution

## Abstract

*Quercus gilva* is an ecologically and economically important species of *Quercus* section *Cyclobalanopsis* and is a dominant species in evergreen broad-leaved forests in subtropical regions of East Asia. In the present study, we reported a high-quality chromosome-scale genome assembly of *Q. gilva*, the first reference genome for section *Cyclobalanopsis*, using the combination of Illumina and PacBio sequencing with Hi-C technologies. The assembled genome size of *Q. gilva* was 889.71 Mb, with a contig number of 773 and a contig N50 of 28.32 Mb. Hi-C scaffolding anchored 859.07 Mb contigs (96.54% of the assembled genome) onto 12 pseudochromosomes, with a scaffold N50 of 70.35 Mb. A combination of *de novo*, homology-based, and transcript-based predictions predicted a final set of 36,442 protein-coding genes distributed on 12 pseudochromosomes, and 97.73% of them were functionally annotated. A total of 535.64 Mb (60.20%) of repetitive sequences were identified. Genome evolution analysis revealed that *Q. gilva* was most closely related to *Q. suber* and they diverged at 40.35 Ma, and *Q. gilva* did not experience species-specific whole-genome duplication in addition to the ancient *gamma* (γ) whole-genome triplication event shared by core eudicot plants. *Q. gilva* underwent considerable gene family expansion and contraction, with 598 expanded and 6,509 contracted gene families detected. The first chromosome-scale genome of *Q. gilva* will promote its germplasm conservation and genetic improvement and provide essential resources for better studying the evolution of *Quercus* section *Cyclobalanopsis*.

## Introduction

The genus *Quercus*, comprising approximately 450 species, is one of the most dominant woody genera throughout Asia, Europe, and America (Plomion et al., 2018; Zhou et al., 2022). Classifying *Quercus* genus has been historically challenging due to low interspecific differentiation and high intraspecific genetic variation partly caused by hybridization and introgression (Hipp, 2015). According to the recent molecular evidence provided by nuclear ribosomal internal transcribed spacer (ITS), restriction fragment length polymorphism (RFLP), and genomics datasets, the species in genus *Quercus* have been grouped into two subgenera *Quercus* and *Cerris* (Manos et al., 2001; Denk and Grimm, 2010; Denk et al., 2017; Deng et al., 2018; Hipp et al., 2020). Subgenus *Quercus*, which is primarily distributed in North America, includes five sections: *Lobatae*, *Ponticae*, *Protobalanus*, *Quercus*, and *Virentes*. Subgenus *Cerris*, of which the major distribution region is Eurasia, consists of *Cerris*, *Cyclobalanopsis*, and *Ilex* sections (Denk et al., 2017).

*Quercus* section *Cyclobalanopsis* is mainly distributed in subtropical and tropical regions of Asia (Denk and Grimm, 2010). Approximately 90 species have been recognized in section *Cyclobalanopsis* and are well-adapted to warm and humid climates. Due to their ecological importance, the phylogeny of *Cyclobalanopsis* species has been investigated using both phenotypic and molecular data over the past ten years (Denk and Grimm, 2010; Deng et al., 2013; Deng et al., 2014; Deng et al., 2018; Hipp et al., 2020), which helped us to better understand the evolutionary history of section *Cyclobalanopsis*. In the latest study, Deng et al. (2018) utilized restriction-site associated DNA sequencing (RAD-seq) data to resolve phylogenetic relationships of 34 *Cyclobalanopsis* species, inferring two major lineages that are compound trichome bases (CTB) lineage and single-celled trichome bases (STB) lineage. RAD-seq is a fractional genome sequencing strategy that usually only samples a small proportion of the genome (Davey and Blaxter, 2010; Lowry et al., 2017). Moreover, the RAD-seq approach relies on enzymes to isolate restriction site fragments; the polymorphic sites occurring at restriction sites consequently lead to missing information, resulting in potential bias in the phylogenetic estimation (Ai et al., 2022). The genome-wide sequencing data of white oaks (e.g., *Q. robur*, *Q. lobata*, *Q. mongolica*) have contributed significantly to resolving phylogenetic relationships within section *Quercus* (Plomion et al., 2018; Ai et al., 2022; Sork et al., 2022). Therefore, the availability of whole-genome sequencing data for *Cyclobalanopsis* species is essential to the phylogenetic inference of section *Cyclobalanopsis*. However, none of *Cyclobalanopsis* species have available genome-wide data yet.

**Figure 1:**
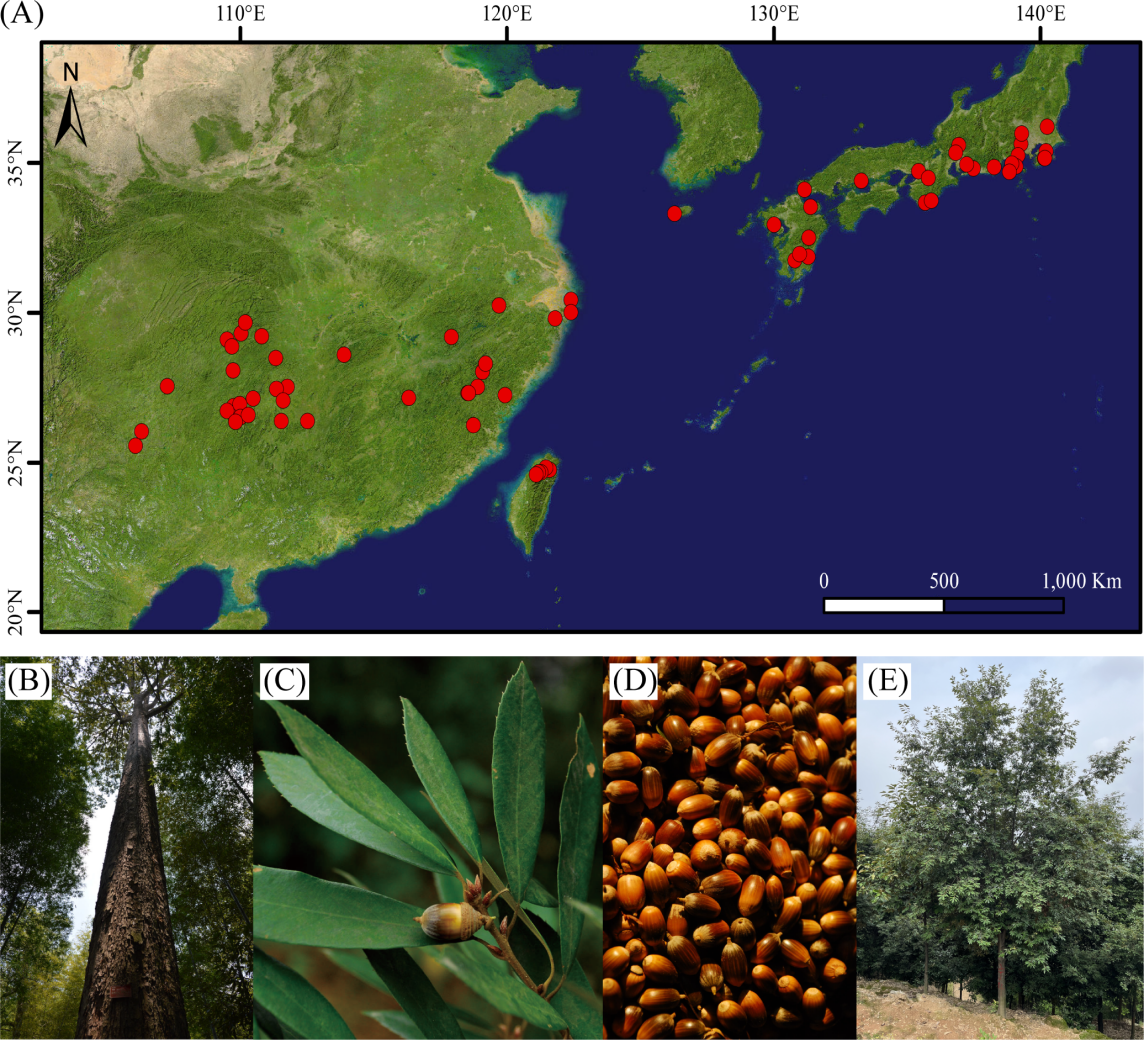
Distribution and characteristics of *Quercus gilva*. **(A)** Distribution of *Q. gilva* based on specimen records, literature, and field survey, **(B)** trunk, **(C)** leaves, and **(D)** fruits of *Q. gilva*, **(E)** individual of the sampled 12-year-old *Q. gilva*.

*Quercus gilva* (2n=2x=24) is a representative species of section *Cyclobalanopsis* in East Asia, including southern and southeastern China, Japan, and Jeju Island of South Korea (Zeng et al., 2019; Han et al., 2020) (**Figure 1A**). *Q. gilva* is a native and dominant species in evergreen broad-leaved forests in subtropical areas of East Asia. In China, *Q. gilva* naturally distributes in mixed and secondary forests from 106°-122°E to 22°-29°N at altitudes of 300-1500 m (Zeng et al., 2019). It provides essential ecological services, including water conservation, soil protection, and carbon sequestration. Besides, its red, hard, and well-textured heartwood provides quality materials for high-end furniture and fine artware production (Zeng et al., 2019) (**Figures 1B–D**). *Q. gilva* has been therefore considered to be ecologically and economically valuable. However, the once widespread *Q. gilva* populations have greatly diminished as a consequence of human disturbance (e.g., large-scale logging and regional development) (Deng et al., 2018). Limited studies have been conducted on *Q. gilva*, which focused on its identification (Ohyama et al., 2001; Noshiro and Sasaki, 2011), marker development (Sugiura et al., 2014), genetic diversity (Sugiura et al., 2015), and potential distributions (Han et al., 2020). Although a chloroplast genome of *Q. gilva* was reported (Zeng et al., 2019), nuclear genome information is not available for *Q. gilva*.

We herein report the first chromosome-scale genome assembly and reveal the genome evolution of *Q. gilva*. This high-quality reference genome will promote germplasm conservation and genetic improvement of *Q. gilva* and provide essential resources for better understanding the phylogenetic relationships of *Quercus* section *Cyclobalanopsis*.

## Materials and methods

### Plant materials and DNA extraction

Fresh leaf tissues were sampled from a 12-year-old *Q. gilva* individual growing in Yuchi State-Owned Forest Farm (113.0697°E, 28.5965°N), Hunan Province, China (**Figure 1E**). Leaves were immediately stored in liquid nitrogen until being transported back to the laboratory and stored at -80°C. The genomic DNA extraction from leaf tissues was performed using DNeasy Plant Mini Kit (QIAGEN, Valencia, CA, USA). The quality and quantity of genomic DNA were assessed by NanoDrop 2000 spectrophotometer (Thermo Fisher Scientific, Wilmington, DE, USA), 0.8% agarose gel electrophoresis, and Qubit 3.0 fluorometer (Life Technologies, CA, USA).

### DNA sequencing and data processing

Three different approaches were adopted to perform genomic DNA sequencing. Illumina libraries were constructed with ~350 bp inserts and sequenced on a NovaSeq 6000 platform (Illumina, San Diego, CA, USA) with paired-end reads of 150 bp (PE 150 bp). Paired-end adapters were removed from raw data using FastUniq v1.1 (Xu et al., 2012). Raw data were then filtered according to the following criteria: (a) duplicate read pairs; (b) reads with unknown bases ≥ 10%; (c) reads of which ≥ 50% bases with Phred quality score ≤ 5. BWA-MEM v0.7.12 (Li and Durbin, 2009) was used to filter out contamination reads. After quality control, Illumina clean reads were obtained and utilized to perform a genome survey.

PacBio library was prepared using the “Procedure & Checklist – Preparing HiFi SMRTbell^®^ Libraries using the SMRTbell Express Template Prep Kit 2.0” protocol (Pacific Biosciences of California, Inc., CA, USA). HiFi sequencing was carried out on a PacBio Sequel II (Pacific Biosciences of California, Inc., CA, USA) with circular consensus sequencing (CCS) mode using Sequel II Binding Kit 2.2 and Sequel II Sequencing Kit 2.0. After removing adapters and low-quality reads using the PacBio SMRT Analysis module in SMRT Link v11.0, HiFi CCS clean data were obtained and used for subsequent analyses.

A Hi-C library was generated following the approach described by Lieberman-Aiden et al. (2009). Briefly, chromatin was fixed, extracted, and digested. Subsequently, DNA was purified from protein, then randomly sheared into fragment sizes of 100-500 bp, and sequenced using PE 150 bp mode on a NovaSeq 6000 platform. Data were cleaned and processed in the same manner as described in the genomic DNA sequencing for the genome survey.

### RNA extraction and sequencing

Total RNA was extracted from root, leaf, and branch tissues of the same *Q. gilva* individual that was used for genome sequencing using RNAprep Pure Plant Kit (Tiangen, Beijing, China). RNA Nano 6000 Assay Kit of Agilent Bioanalyzer 2100 system (Agilent Technologies, CA, USA) and NanoDrop 2000 spectrophotometer (Thermo Fisher Scientific, Wilmington, DE, USA) were then used to examine RNA quality and quantity. A mixture of total RNA from three tissues was used for library construction, performed by Hieff NGS Ultima Dual-mode mRNA Library Prep Kit for Illumina (Yeasen Biotechnology, Shanghai, China) and sequenced using an Illumina NovaSeq 6000 platform with PE 150 bp mode.

### Genome survey, assembly and assessment

Before *de novo* genome assembly, the genome size, repeated sequences, and heterozygosity were estimated using K-mer analysis with Illumina clean reads. The iterative selection of 19 bp base sequences (K-value=19) was used for K-mer analysis. K-mer frequency distribution was tallied and the K-mer depth distribution curve was calculated, which were then used to evaluate genome size, the percentage of repeated sequences, and heterozygosity ratio as described by Li et al. (2019).

HiFi CCS clean reads were initially assembled using Hifiasm v0.15.4 (Cheng et al., 2021) with parameters -z20 to trim both ends of reads by 20 bp. BWA-MEM v0.7.12 (Li and Durbin, 2009) was then used to align the Hi-C clean data onto the assembled genome. The read pairs were independently mapped to the genome assembly and only read pairs that were uniquely mapped were used in subsequent analyses. Valid interaction paired-end reads were used to cluster, sort, and correct the contigs into 12 pseudochromosomes using 3D-DNA (Dudchenko et al., 2017) and manual inspection was performed with Juicebox v1.22 (Robinson et al., 2018).

The quality of the assembled *Q. gilva* genome was assessed from four aspects. First, Illumina clean reads and CCS clean reads were re-aligned onto the final assembly by BWA-MEM v0.7.12 (Li and Durbin, 2009) and Minimap2 v2.24 (Li, 2018), respectively. Second, BUSCO v5.2.2 assessment (Simão et al., 2015) was performed using the embryophyta_odb10 dataset and default parameters. Third, the long terminal repeat (LTR) Assembly Index (LAI) was applied to assess the assembly continuity as described previously (Ou et al., 2018). Finally, the occurrence of telomeric tandem repeat ((TTAGGG/CCCTAA)n) was examined on both edges of 12 pseudochromosomes to assess the completeness and accuracy of our chromosomal assembly.

### Genome annotation

The repetitive sequences that include tandem repeats and transposable elements (TEs) in the *Q. gilva* genome were identified. Tandem repeats were annotated using MISA v2.1 (Thiel et al., 2003). TEs in the assembled genome were identified using *de novo* and homology-based approaches. A *de novo* repetitive sequence library of the *Q. gilva* genome was constructed using RepeatModeler v2.0 (http://www.repeatmasker.org/RepeatModeler/) and TEs were subsequently identified using RepeatMasker v4.0.5 (Tarailo-Graovac and Chen, 2009). For the homology-based approach, the assembled *Q. gilva* genome was aligned against the Repbase database v20.05 (Bao et al., 2015) using RepeatMasker v4.0.5 with default parameters for TEs identification. Results from these two approaches were merged to yield final TEs in the assembled *Q. gilva* genome.

A combination strategy of *de novo*, homology-based, and transcript-based predictions was applied to predict protein-coding genes. *De novo* prediction was performed using AUGUSTUS v3.3.3 (Stanke et al., 2004) on the repeat-masked sequences. In homology-based prediction, the protein sequences of *Quercus aquifolioides*, *Quercus lobata* (Sork et al., 2022), *Quercus mongolica* (Ai et al., 2022), *Quercus robur* (Plomion et al., 2018), and *Quercus suber* (Ramos et al., 2018) were aligned against the *Q. gilva* assembly using TBLASTN v2.60 (Gertz et al., 2006). GeMoMa v1.8 (Keilwagen et al., 2016) was then employed to predict protein-coding genes based on homologous sequences. In the transcript-based approach, RNA sequencing clean data were mapped to the *Q. gilva* genome by HISAT2 v2.2.0 (Kim et al., 2019), and transcripts were then assembled using StringTie v2.1.3 (Pertea et al., 2015). PASA v2.4.1 (Haas et al., 2003) was utilized to predict gene models. MAKER v3.1.2 (Cantarel et al., 2008) was used to integrate the results from *de novo*, homology-based, and transcript-based approaches to generate a consensus gene set.

Functional annotation of the predicted genes was performed by searching for the best matches of alignments in non-redundant (NR) (https://ftp.ncbi.nlm.nih.gov/blast/db/FASTA/), Swiss-prot (Boeckmann et al., 2003), and Eukaryotic Orthologous Groups (KOG) (Tatusov et al., 2003) using BLASTP v.2.7.1 (Camacho et al., 2009) with e-value ≤ 1e^–5^. Gene Ontology (GO) (Ashburner et al., 2000) terms were assigned to the predicted genes based on eggNog-mapper v2.1.6 (Cantalapiedra et al., 2021) annotation. Putative gene pathways were inferred based on the Kyoto Encyclopedia of Genes and Genomes (KEGG) databases using the BlastKOALA webservice (http://www.kegg.jp/blastkoala/) (Kanehisa et al., 2016). Protein domains and motifs were characterized using InterProScan v5.42-78.0 (Jones et al., 2014) with Pfam (Finn et al., 2014) database.

Non-coding RNAs (ncRNAs), which include ribosomal RNAs (rRNAs), transfer RNAs (tRNAs), micro RNAs (miRNAs), and small nuclear RNAs (snRNAs), were identified through sequence alignment to the Rfam database (Griffiths-Jones et al., 2005). The rRNAs were identified using RNAmmer v1.2 (Lagesen et al., 2007). The tRNAs were predicted using tRNAscan-SE v1.3.1 (Schattner et al., 2005) with eukaryote parameters. Infernal v1.1 (Nawrocki and Eddy, 2013) was used to detect miRNAs and snRNAs.

### Phylogenetic analysis and divergence time estimation

Protein sequences of *Arabidopsis thaliana, Betula platyphylla, Castanea dentata, Fagus sylvatica, Oryza sativa, Populus trichocarpa, Q. gilva, Q. lobata, Q. suber*, and *Ricinus communis* were used to cluster the orthologous genes. For each gene, only the longest transcript was retained and protein sequences that are less than 50 amino acids in length or have internal stop codons were filtered. Gene family clustering analysis was then performed using OrthoFinder v2.5.2 (Emms and Kelly, 2019) with filtered protein sequences of the 10 species.

A phylogenetic tree on a basis of shared single-copy orthologous genes was generated for *A. thaliana*, *B. platyphylla*, *C. dentata*, *F. sylvatica*, *O. sativa*, *P. trichocarpa*, *Q. gilva*, *Q. lobata*, *Q. suber*, and *R. communis*. MAFFT v7.490 (Katoh and Standley, 2013) was used to independently perform the multiple sequence alignment for each gene, and Gblocks v0.91b (Talavera and Castresana, 2007) was then utilized to filter poorly aligned sequences. Protein sequences of all single-copy orthologous genes were concatenated, which was then used to construct a maximum-likelihood (ML) tree by RAxML v8.2.12 (Stamatakis, 2014) with PROTGAMMALGX model of sequence evolution.

Divergence times between *Q. gilva* and nine other species were estimated with MCMCTree in PAML v4.10.0 (Yang, 2007). Two calibration nodes were used in divergence time estimation. The first calibration was the divergence time between *A. thaliana* and *O. sativa* (152 Ma) obtained from the TimeTree database (http://www.timetree.org) (Kumar et al., 2017), which is widely used to estimate divergence times among plant species. The pollen fossil of a *Quercus* specimen (Hofmann et al., 2011) that has been commonly used to constrain the node of genus *Quercus* (Hipp et al., 2020; Zhou et al., 2022) was used to calibrate the stem node of *Q. gilva*, *Q. suber*, and *Q. lobata* with 56 (54-60) Ma.

### Gene family contraction and expansion analysis

To examine gene family expansion and contraction between the ancestor and each species, gene family clustering and phylogenetic analysis results were inputted into CAFÉ v3.1 (De Bie et al., 2006). Significant gene family expansion and contraction were determined with *P*-value ≤ 0.05. Functional enrichment analysis was conducted to identify expanded and contracted gene families in the *Q. gilva* genome. GO term assignment and KEGG pathway analysis were performed using eggNog-mapper v2.1.6 (Cantalapiedra et al., 2021) and BlastKOALA webservice (Kanehisa et al., 2016), respectively.

### Genome synteny and whole-genome duplication analysis

Colinear maps were generated by comparing genome sequences on 12 pseudochromosomes of *Q. gilva* with *Q. lobata* and *Q mongolica* genomes using MUMmer v4.0 (Marçais et al., 2018) to investigate the syntenic relationship between *Q. gilva* and these two oak genomes. Additionally, synteny analysis was performed between *Q. gilva* and *Q. lobata* genomes and between *Q. gilva* and *Q. mongolica* genomes using JCVI v1.1.19 (Tang et al., 2008) with following parameters: – cscore=.99, –minspan=30. The block comprising at least five sequential genes and with C-score≥0.99 was defined as the initial syntenic block and the syntenic blocks spanning more than 30 genes were displayed in the synteny map. According to the previous findings that *Q. lobata* does not undergo species-specific whole-genome duplication (WGD) events besides the ancient *gamma* whole-genome triplication (γ-WGT) event shared by core eudicot plants (Ai et al., 2022), *Q. lobata* and *Q. suber* were selected for the inference of WGD events in *Q. gilva*. Protein sequences of these species were compared with *Q. gilva* genome to identify syntenic blocks and syntenic genes using BLASTP v.2.7.1 (Camacho et al., 2009) (e-value ≤ 1e^–5^). Synonymous substitution rate (Ks) of the syntenic gene pairs within and among genomes was calculated using KaKs_Calculator 2.0 (Wang et al., 2010) and ParaAT v2.0 (Zhang et al., 2012), respectively. The probability density distribution curve of Ks was visualized using R software, and WGD events were inferred from the distribution peaks.

## Results

### Genome survey, assembly and assessment

A genome survey was performed to predict the genome size, repeated sequences, and heterozygosity of *Q. gilva* using K-mer analysis based on ~55.66 Gb of Illumina clean data (**Supplementary Table 1**). With a K-mer number of 47,492,571,457 and K-mer depth of ~54.86, the genome size was estimated to be ~865.75 Mb. A high level of heterozygosity ratio of 1.16% and ~48.17% of repeated sequences were observed (**Supplementary Figure 1**; **Supplementary Table 2**).

**Figure 2:**
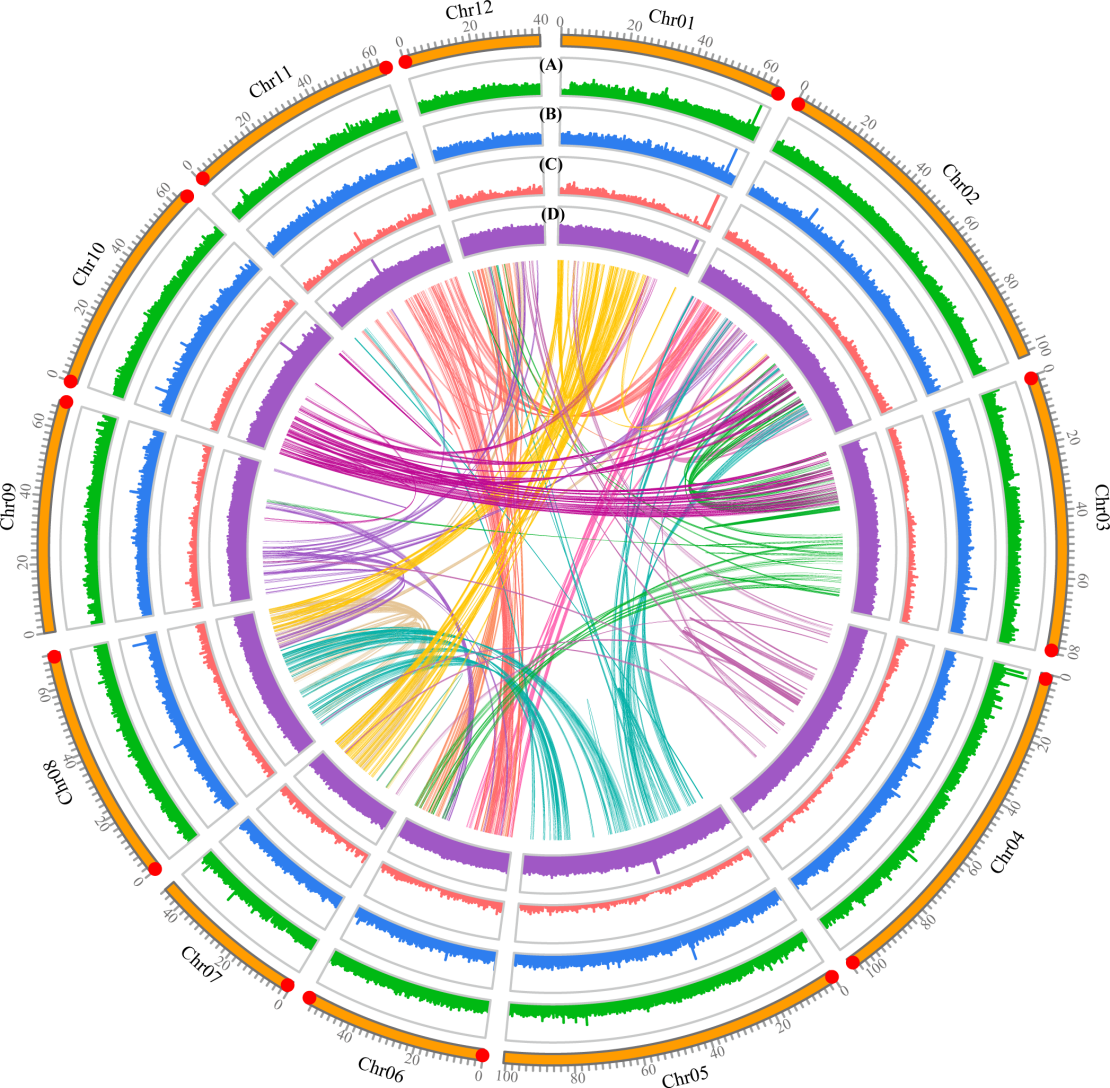
Features of the *Quercus gilva* genome. The outermost circle represents the 12 pseudochromosomes, with red dot at the end indicating telomeric repeat ((TTAGGG/CCCTAA)n) reached. From outer to inner circles: **(A)** sequence coverage by PacBio CSS clean reads; **(B)** LTR Assembly Index (LAI); **(C)** gene density; **(D)** GC content. **(A–D)** were drawn in 50 kb non-overlapping sliding windows. The intragenomic syntenic blocks were indicated by the innermost circle.

A total of ~30.76 Gb of HiFi CCS clean reads were produced by the PacBio Sequel II and used for the subsequent genome assembly (**Supplementary Table 1**). The contigs were then polished with HiFi CCS clean data, generating a genome assembly of 889.71 Mb, with a number of contigs of 773 and a contig N50 of 28.32 Mb (**Table 1**). The contig N50 of the assembled *Q. gilva* genome is ~11-fold and ~405-fold compared with *Q. mongolica* and *Q. robur*, respectively. In total, ~121.70 Gb of Hi-C data were obtained and connected to 12 pseudochromosomes (**Supplementary Table 1**). Finally, 859.07 Mb of sequences (96.54% of the genome assembly) were anchored onto 12 pseudochromosomes, ranging in sizes of 40.26-104.15 Mb (**Figure 2**; **Supplementary Figure 2**; **Supplementary Table 3**). The chromosome-scale genome assembly of *Q. gilva* was characterized by a scaffold number of 515 and a scaffold N50 of 70.35 Mb. The scaffold N50 of *Q. gilva* is similar to that of *Q. mongolica* (66.74 Mb) and *Q. lobata* (75.00 Mb) while ~53-fold than *Q. robur* (1.34 Mb).

**Table 1:** Comparison of genome assembly and annotation between *Quercus* species.

	*Q. gilva*	*Q. lobata*	*Q. mongolica*	*Q. robur*
Sequencing platform	Illumina, PacBio, Hi-C	Illumina, PacBio, Hi-C	Illumina, PacBio, Hi-C	Illumina, Roche 454
Assembly				
Assembly version	This study	ValleyOak v3.0	*Quercus mongolica* v1	Haploid v2
Number of contigs	773	*	645	22,615
Total contig length (Mb)	890	*	810	790
Contig N50 size (Mb)	28.32	*	2.64	0.07
Number of scaffolds	515	2,010	330	1,409
Total scaffold length (Mb)	890	846	810	814
Scaffold N50 size (Mb)	70.35	75.00	66.74	1.34
% of sequence anchored on chromosome	97	96	96	96
Annotation				
Number of protein-coding genes	36,442	39,373	36,553	25,808
Average length of gene (kb)	3.7	5.4	6.1	2.9
Average length of CDS (kb)	1.0	1.3	1.2	1.2
Average exons per gene	4.5	5.5	4.8	*

Information of Q. lobata, Q. mongolica, and Q. robur was referenced from the published articles (Plomion et al., 2018; Ai et al., 2022; Sork et al., 2022).

*Data were not provided in the original articles.

The quality of the *Q. gilva* genome assembly was assessed by four approaches. First, 100% of Illumina short reads and 99.85% of CCS clean reads were mapped to the assembled genome, which could cover 96.22% and 99.87% of the assembled genome sequence, respectively (**Figure 2**; **Supplementary Table 4**). Second, among 1,614 Benchmarking Universal Single-Copy Orthologs (BUSCO) genes, 98.6% of complete BUSCOs, including 93.5% of complete single-copy and 5.1% of complete duplicated, were detectable in our assembled genome (**Supplementary Figure 3**). Third, LAI of the *Q. gilva* genome assembly is 22.71 which is greater than the gold standard quality level of the assembly (LAI=20) (Ou et al., 2018) (**Supplementary Figure 4**). Additionally, our chromosomal assembly reached telomeric tandem repeats on both ends of six pseudochromosomes and on one end of six more (**Figure 2**). These results elucidated the high completeness and quality of our *Q. gilva* genome assembly.

### Genome annotation

In total, 929,678 tandem repeats, including 663,186 mono-, 199,396 di-, 53,203 tri-, 10,358 tetra-, 2,064 penta-, and 1,471 hexa-nucleotide repeats, were identified in the *Q. gilva* genome, accounting for ~2.63% of the assembled genome (23.38 Mb) (**Supplementary Table 5**). Approximately 512.26 Mb (~57.57% of the genome) of TEs were identified *via* the combination of *de novo* and homology-based predictions, with 0.20% of short interspersed nuclear elements (SINEs), 3.89% of long interspersed nuclear elements (LINEs), 18.67% of long terminal repeats (LTRs), and 2.41% of DNA transposons. The percentage of TEs in *Q. gilva* is comparable to that of *Q. lobata* (54.4%) and *Q. robur* (53.3%).

A final set of 36,442 protein-coding genes distributed on 12 pseudochromosomes was predicted through a combination of *de novo*, homology-based, and transcript-based approaches, with average exons per gene of 4.5 (**Table 1**; **Supplementary Figure 5**; **Supplementary Table 6**). The average gene and CDS length were 3,724 and 980 bp, respectively. In total, ~97.73% of the predicted protein-coding genes (35,615 genes) were functionally annotated in the databases described above (**Supplementary Figure 6**; **Supplementary Table 7**). The ncRNAs were identified in the *Q. gilva* genome, which included 709 tRNAs, 1,798 rRNAs, 38 miRNAs, and 142 snRNAs.

### Phylogenetic analysis

**Figure 3:**
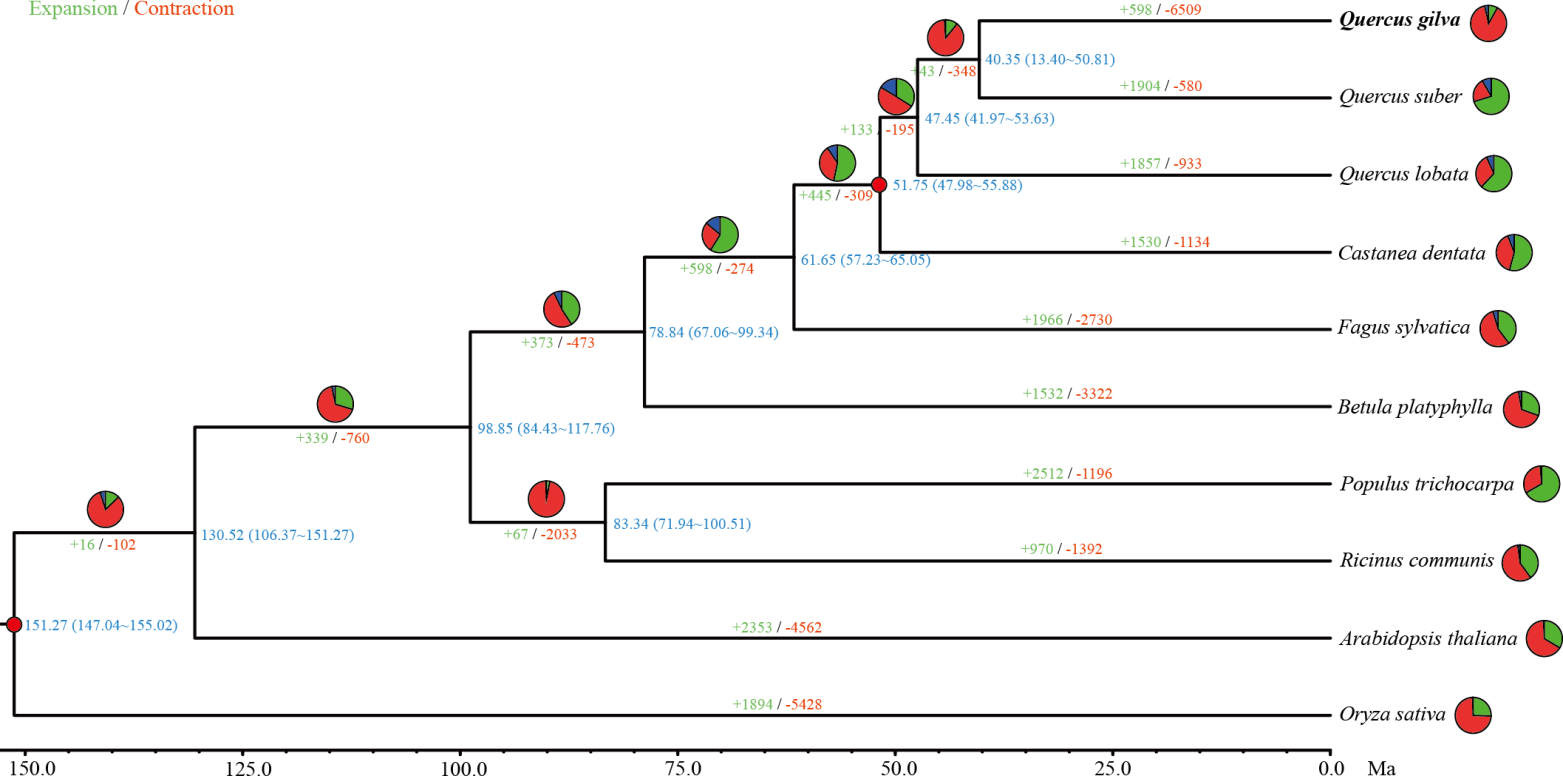
Phylogenetic tree based on shared single-copy gene families among *Quercus gilva* and nine other species. Inferred maximum-likelihood (ML) phylogenetic tree was generated on the basis of 1,244 single-copy orthologous genes across 10 species. The numerical value at the right of each node shows the estimated divergence time in millions of years. Red dots indicate calibrated nodes. Numbers in green (+) and red (−) show the number of expanded and contracted gene families, respectively. The green, red, and blue portions of the pie charts indicate the percentage of gene families undergoing expansion, contraction, and rapidly evolving event, respectively.

Gene family clustering analysis assigned 235,227 genes from *Q. gilva* and nine other species to 20,844 orthogroups. A total of 13,241 genes clustered into 9,259 gene families were revealed in *Q. gilva* genome through comparisons of protein sequences homologous between *Q. gilva* and nine other species (**Supplementary Figure 7**; **Supplementary Table 8**). In total, 1,244 single-copy orthologous genes were shared among *Q. gilva* and nine other species, which were used to construct a phylogenetic tree and to estimate species divergence time (**Figure 3**).

The maximum-likelihood phylogenetic tree indicated that *Q. gilva* (in section *Cyclobalanopsis*) was most closely related to *Q. suber* (in section *Cerris*), with a divergence time at ~40.35 (13.40-50.81) Ma. The estimated divergence time of *Q. lobata* (species of subgenus *Quercus*) from the common ancestor of *Q. gilva* and *Q. suber* (members of subgenus *Cerris*) was ~47.45 (41.97-53.63) Ma. The estimated split between *C. dentata* and three *Quercus* species was ~51.75 (47.98-55.88) Ma.

### Gene family contraction and expansion

In total, 598 expanded and 6,509 contracted gene families have been observed in the *Q. gilva* genome (**Figure 3**). Among them, 41 and 207 gene families were significantly expanded and contracted (*P*<0.05), respectively. The expanded gene families in the *Q. gilva* were significantly enriched in 565 GO terms (*Q*<0.01), which were primarily enriched in cellular component of cell (GO:0005623, 59 genes) and molecular function of catalytic activity (GO:0003824, 52 genes) (**Supplementary Table 9**). The contracted gene families showed significant enrichment in 398 GO terms, with major enrichment in biological process of cellular process (GO:0009987, 37 genes) and cellular component of cell part (GO:0044464, 37 genes) (**Supplementary Table 10**). KEGG enrichment analysis of expanded gene families revealed only two significantly enriched pathways (*Q*<0.01) (**Supplementary Table 11**). While the contracted gene families were found to be significantly enriched in 15 KEGG pathways, with the chief enrichment in plant-pathogen interaction (ko04626) (**Supplementary Table 12**).

### Genome synteny and whole-genome duplication

**Figure 4:**
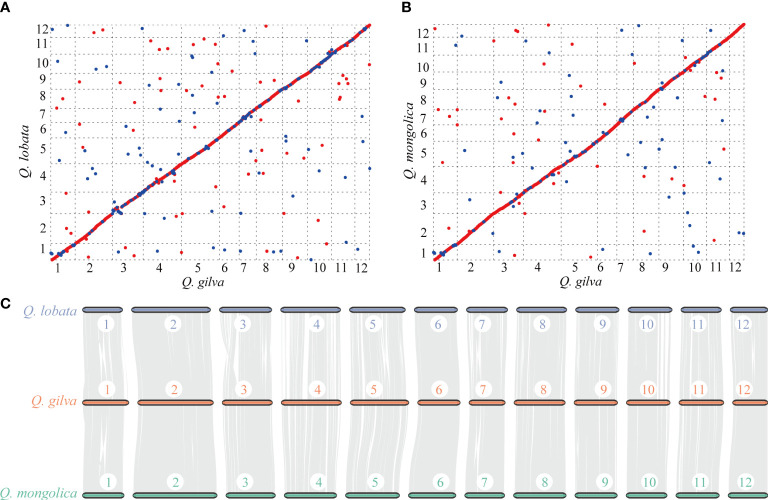
Syntenic analysis through comparisons of the 12 pseudochromosomes of *Q. gilva* with *Q. lobata* and *Q. mongolica*. **(A)** Colinear map of the *Q. gilva* and *Q. lobata* genomes. **(B)** Colinear map of the *Q. gilva* and *Q. mongolica* genomes. Red and blue dots indicate the identical sequence in the same and opposite orientation, respectively. **(C)** Chromosome-level syntenic comparisons based on gene pairs between *Q. gilva* and *Q. lobata* and between *Q. gilva* and *Q. mongolica*. Syntenic blocks with more than 30 genes are connected by grey lines.

Colinear maps were generated by comparing *Q. gilva* genome with *Q. lobata* (**Figure 4A**) and *Q. mongolica* (**Figure 4B**) genomes. Both maps showed a small proportion (6.4% between *Q. gilva* and *Q. mongolica* genomes and 12.2% between *Q. gilva* and *Q. lobata* genomes) of blue dots showing the identical sequence in the opposite orientation, which elucidated high similarity between *Q. gilva* and *Q. lobata* genomes and between *Q. gilva* and *Q. mongolica* genomes. Moreover, syntenic blocks were generated for *Q. gilva* versus *Q. lobata* genomes and *Q. gilva* versus *Q. mongolica* genomes (**Figure 4C**). In total, 174 and 104 syntenic blocks have been obtained from the comparison of *Q. gilva* versus *Q. lobata* genomes and *Q. gilva* versus *Q. mongolica* genomes, respectively. A one-to-one corresponding relationship of the 12 chromosomes was observed between *Q. gilva* and *Q. lobata* genomes and between *Q. gilva* and *Q. mongolica* genomes.

The distribution curves of Ks for *Q. gilva* and *Q. lobata* showed a peak at ~1.3 Ks units (**Supplementary Figures 8, 9**), indicating these two species shared a WGD event that is *gamma* whole-genome triplication (γ-WGT) event in the common ancestor of core eudicots. Another peak at low values of Ks (0.1-0.2) nearly fitted with exponential distribution was observed in three *Quercus* species, which indicated the recent burst of local gene duplications. The peak value of orthologous gene pairs of *Q. gilva* versus *Q. lobata* and *Q. gilva* versus *Q. suber* (Ks value of 1.1) was lower than the peak value of paralogous gene pairs of *Q. gilva* and *Q. lobata*, implying that the divergence between *Q. gilva* and two other *Quercus* species occurred later than the shared γ-WGT and *Q. gilva* did not experience species-specific WGD events.

## Discussion

In the present study, a high-quality chromosome-scale genome assembly of *Q. gilva* was generated by employing a combination strategy of Illumina NovaSeq 6000, PacBio Sequel II, and Hi-C sequencing technologies. The assembled genome size of *Q. gilva* was ~890 Mb, with contig and scaffold N50 values of 28.32 and 70.35 Mb, respectively. Greater than 96% of the *Q. gilva* genome sequences (~859 Mb) were anchored onto the 12 pseudochromosomes that ranged in a size of 40.26-104.15 Mb. Consistent with *Q. lobata* and *Q. mongolica* genomes, the greatest number of genes were observed on chromosome 2 (4,774) among the 12 chromosomes of *Q. gilva*. However, differing from *Q. lobata* and *Q. mongolica* genomes that chromosome 2 was found to be the longest, chromosome 4 is slightly longer (104.15 Mb) than chromosome 2 (101.11 Mb) in our *Q. gilva* genome assembly. The increase in the length of chromosome 4 may be caused by the high level of transposable elements (TEs) (~69 Mb) present on it. Genome assembly of woody plants has been generally challenged due to high levels of duplication and heterozygosity. The level of repetitive elements, specifically TEs, was found to be constantly high in genus *Quercus*, for instance, 51.78% in *Q. mongolica* (Ai et al., 2022), 53.3% in *Q. robur* (Plomion et al., 2018), 54.4% in *Q. lobata* (Sork et al., 2022), and 57.57% in *Q. gilva*. The heterozygosity of *Q. gilva* was estimated at 1.16%, which is comparable to *Q. mongolica* (1.09%) (Ai et al., 2022) and *Q. lobata* (1.25%) (Sork et al., 2016). Although high levels of repetitive sequences and heterozygosity are present in *Q. gilva* genome, our assembled genome displays a high assembly quality, with 98.6% of complete BUSCOs detected in the genome assembly, an LAI score of 22.71, and telomeric tandem repeats reaching at both ends of six pseudochromosomes and at one end of six more. This *Q. gilva* genome is the first reference genome for *Quercus* section *Cyclobalanopsis*. It will provide essential information to better understand the evolution of this dominant lineage in East Asia.

Phylogenetic analysis revealed that *Q. gilva* was most related to *Q. suber* (section *Cerris*), with an estimated divergence time of 40.35 (13.40-50.81) Ma, and the estimated split time between two subgenus *Cerris* species (*Q. gilva* and *Q. suber*) and *Q. lobata* (subgenus *Quercus*) was 47.45 (41.97-53.63) Ma. The result is consistent with the phylogenetic structure resolved by previous reports that the divergence of section *Cyclobalanopsis* from sections *Cerris* and *Ilex* and the divergence between subgenera *Cerris* and *Quercus* occurred in the early Eocene (Hipp et al., 2020; Zhou et al., 2022). While our study suggested earlier divergences between *Quercus* species than those proposed by Deng et al. (2018), which may be due to the different fossil calibrations of genus *Quercus* used in this previous study. Analysis of WGD event based on Ks distribution elucidated that *Q. gilva* and *Q. lobata* only underwent the *gamma* whole-genome triplication (γ-WGT) that was shared by core eudicot plants with a Ks peak value of 1.3. A deviated peak value (Ks of 1.1) was observed in *Q. suber*, which supports the previous finding that this deviation may be caused by the low-quality assembly of *Q. suber* using second-generation sequencing (Ai et al., 2022). Moreover, Ks distribution curve elucidated that the divergence between *Q. gilva* and the other two *Quercus* species occurred later than the shared γ-WGT and *Q. gilva* did not experience species-specific WGD events since Ks peak value (1.1) of orthologous gene pairs of *Q. gilva* versus *Q. lobata* and *Q. gilva* versus *Q. suber* was lower than the peak value (1.3) of paralogous gene pairs of *Q. gilva* and *Q. lobata*. The WGD event could also be inferred from the high level of synteny between *Q. gilva* and the other two *Quercus* genomes. A one-to-one corresponding relationship of the 12 chromosomes was found between *Q. gilva* and *Q. lobata* and between *Q. gilva* and *Q. mongolica*. At the same time, fewer inversions occurred between *Q. gilva* and *Q. mongolica* genomes, which may be due to the use of PacBio Sequel II sequencing technology with higher accuracy in *Q. gilva* and *Q. mongolica*. Based on the previous findings that *Q. lobata* and *Q. mongolica* did not experience lineage-specific WGD besides γ-WGT (Ai et al., 2022) and the high collinearity between *Q. gilva* and these two species, we could confirm that no lineage-specific WGD occurred in *Q. gilva*.

Compared with *Q. suber* and *Q. lobata*, the *Q. gilva* genome experienced considerable gene family contraction, with genes related to the plant-pathogen interaction pathway significantly contracted in the *Q. gilva* genome. The previous study indicated that as the key component of plant-pathogen interaction, disease-resistance (R) genes strongly expanded in *Q. robur* (Plomion et al., 2018). Our study may not support this point, however, it is consistent with the finding observed on *Q. mongolica* that the gene families in the plant-pathogen interaction pathway of this Asian oak exhibited significant contraction and the R gene number in *Q. mongolica* was considerably lower compared with other oak species (Ai et al., 2022). In the East Asian environment, the absence of some pathogens may result in a reduced number of corresponding resistance genes from the standpoint of fitness cost, leading to the contraction of related genes (Tian et al., 2003).

In conclusion, we herein report a high-quality chromosome-scale genome assembly of *Q. gilva*, the first reference genome for *Quercus* section *Cyclobalanopsis*, and elucidate the genome evolution of this ecologically and economically important species. Our study will promote germplasm conservation and genetic improvement of *Q. gilva* and provide valuable resources for a better understanding of the evolution of *Quercus* section *Cyclobalanopsis*.

## Data availability statement

The datasets presented in this study can be found in online repositories. The names of the repository/repositories and accession number(s) can be found below: https://www.ncbi.nlm.nih.gov/, PRJNA833760; https://figshare.com/, 10.6084/m9.figshare.20411082.

## Author contributions

HL and FC conceived and designed this research. XZ, NL, XJ, and ZQ conducted the genome data analyses. XZ and NL wrote the manuscript. TF participated in the interpretation and discussion of results and revised the manuscript. All authors have reviewed and approved the final version of this manuscript.

## Funding

This work was funded by National Natural Science Foundation of China (32201589), Natural Science Foundation of Hunan Province (2021JJ41069), and Forestry Scientific and Technological Innovation Project of Hunan Forestry Department (XLKY202218).

## Acknowledgments

We would like to thank Changsha Luo and Tiantian Li for their technical support on genome data analysis.

## Conflict of interest

The authors declare that the research was conducted in the absence of any commercial or financial relationships that could be construed as a potential conflict of interest.

## Publisher’s note

All claims expressed in this article are solely those of the authors and do not necessarily represent those of their affiliated organizations, or those of the publisher, the editors and the reviewers. Any product that may be evaluated in this article, or claim that may be made by its manufacturer, is not guaranteed or endorsed by the publisher.
