# Supplementary material for: A chromosome-scale genome assembly of Quercus gilva: Insights into the evolution of Quercus section Cyclobalanopsis (Fagaceae)
Source: Front Plant Sci. 2022 Sep 23;13:1012277. doi: 10.3389/fpls.2022.1012277 (PMC9539764; doi:10.3389/fpls.2022.1012277)
Supplement: Supplementary file 1 [file DataSheet_1.docx]

Supplementary Material

# Supplementary Figures and Tables

## Supplementary Figures


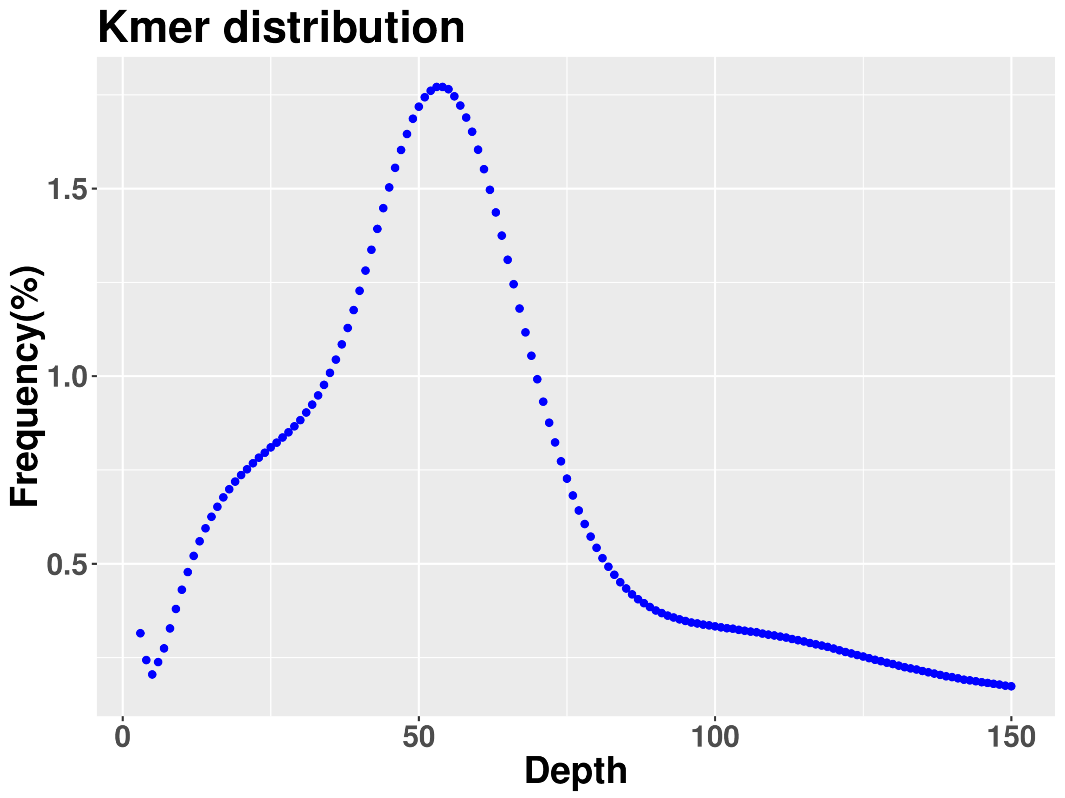


**Supplementary Figure 1.** K-mer analysis of the *Quercus gilva* genome based on Illumina clean data.


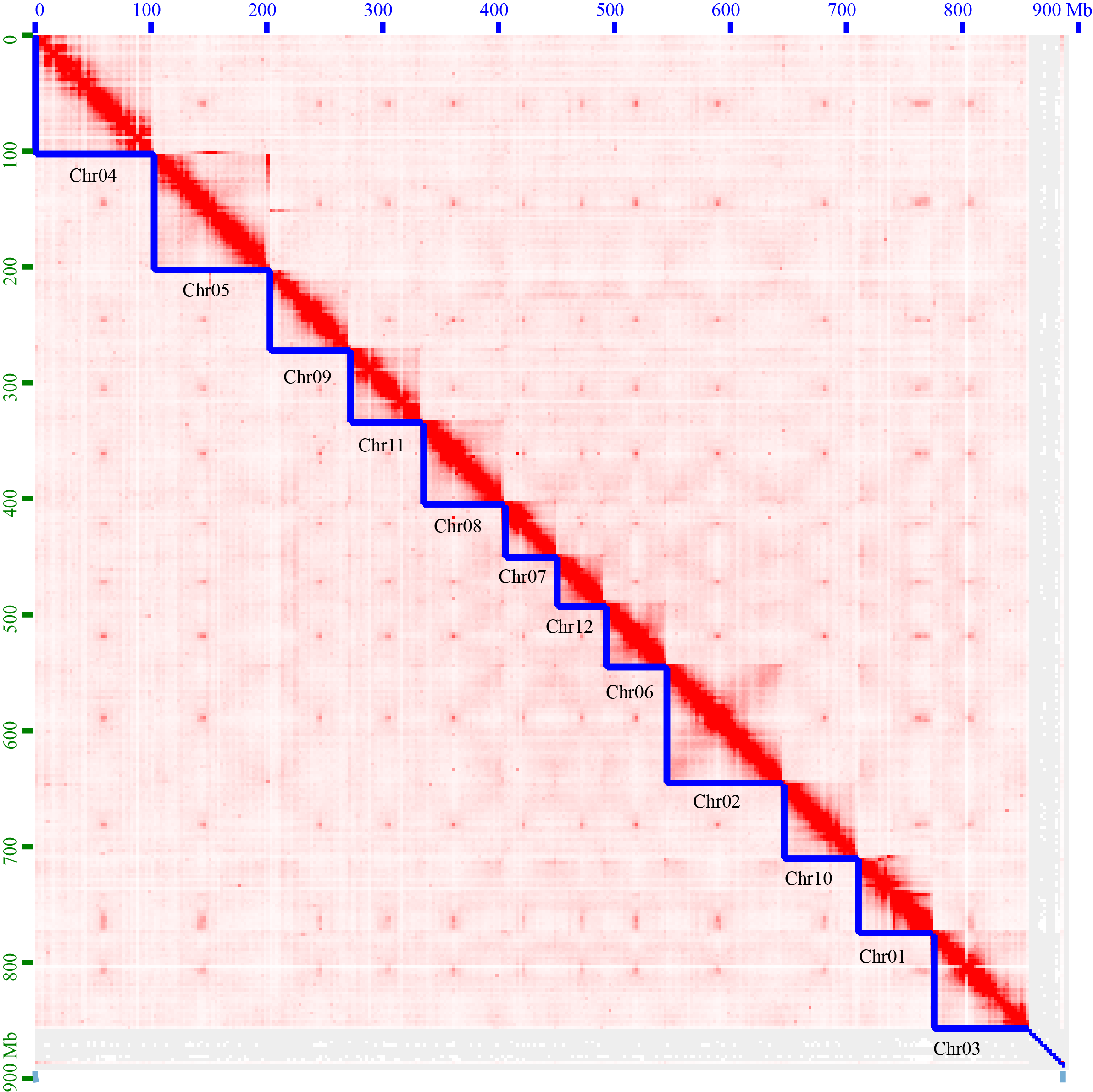


**Supplementary Figure 2.** Hi-C assisted assembly of *Quercus gilva* pseudochromosomes. Heatmap shows Hi-C interactions along the *Q. gilva* genome assembly.

**
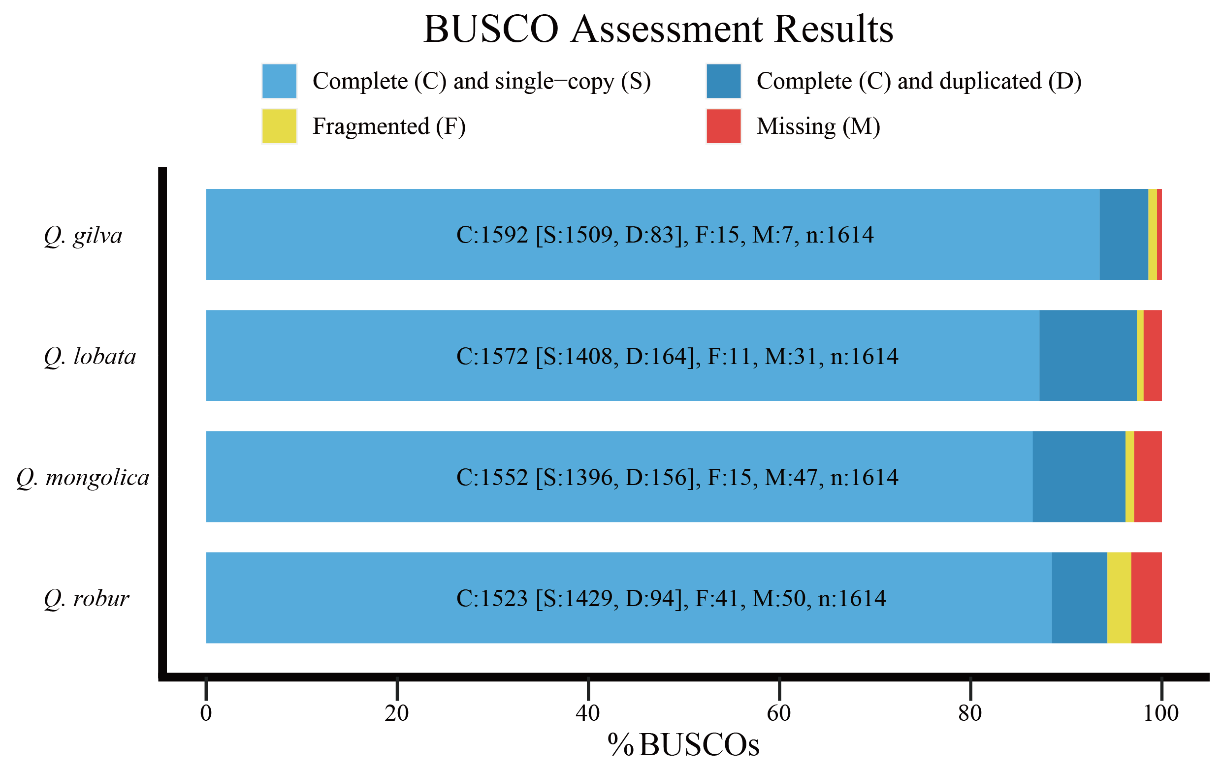
**

Supplementary Figure 3. BUSCO v5.2.2 assessment on genome assembly of *Quercus gilva*, *Q. lobata*, *Q. mongolica* and *Q. robur* against embryophyta_odb10 dataset.


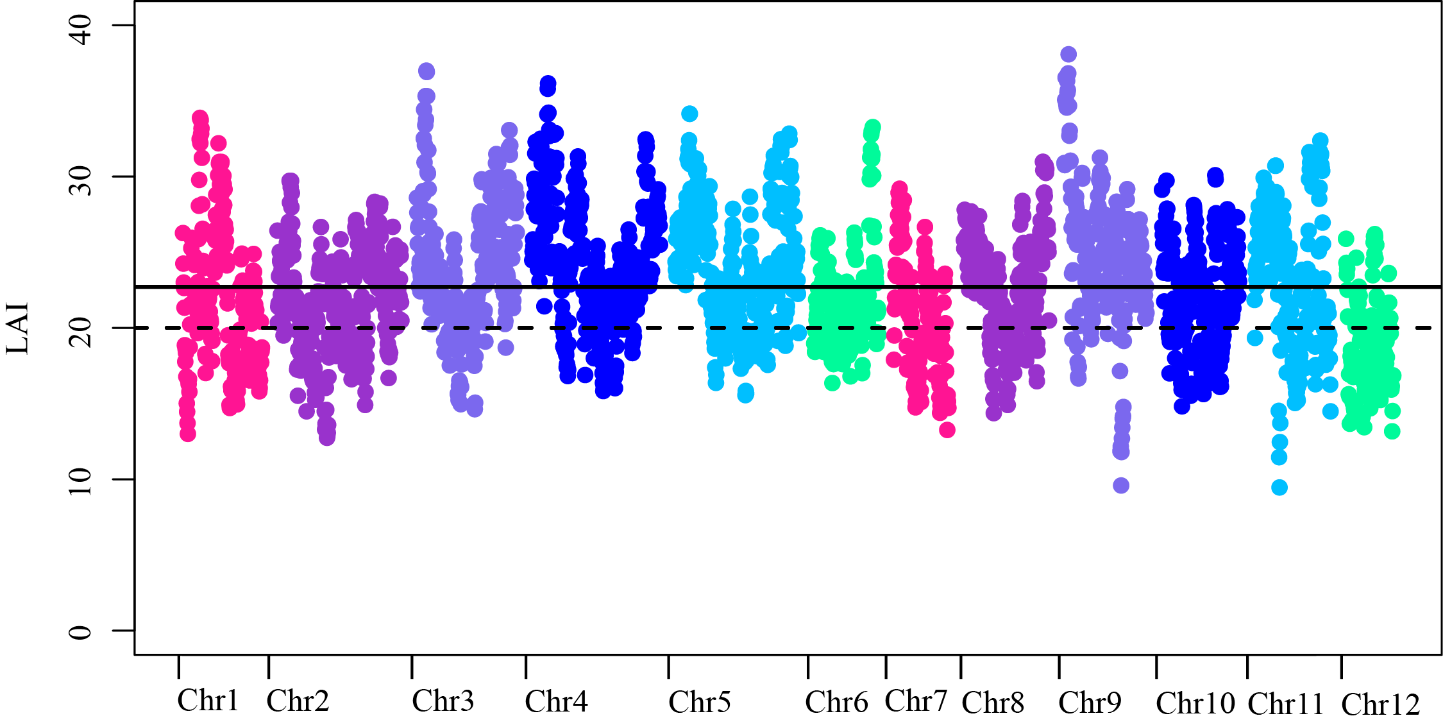


Supplementary Figure 4. LTR Assembly Index (LAI) assessment for assembled pseudochromosomes of *Quercus gilva*. Each dot represents LAI score of a 50 kb nonoverlapping sliding window, which was adjusted by whole-genome LTR identity. Solid line indicates an average LAI of 22.71. The gold standard quality level of the assembly (LAI = 20) is shown by dash line.


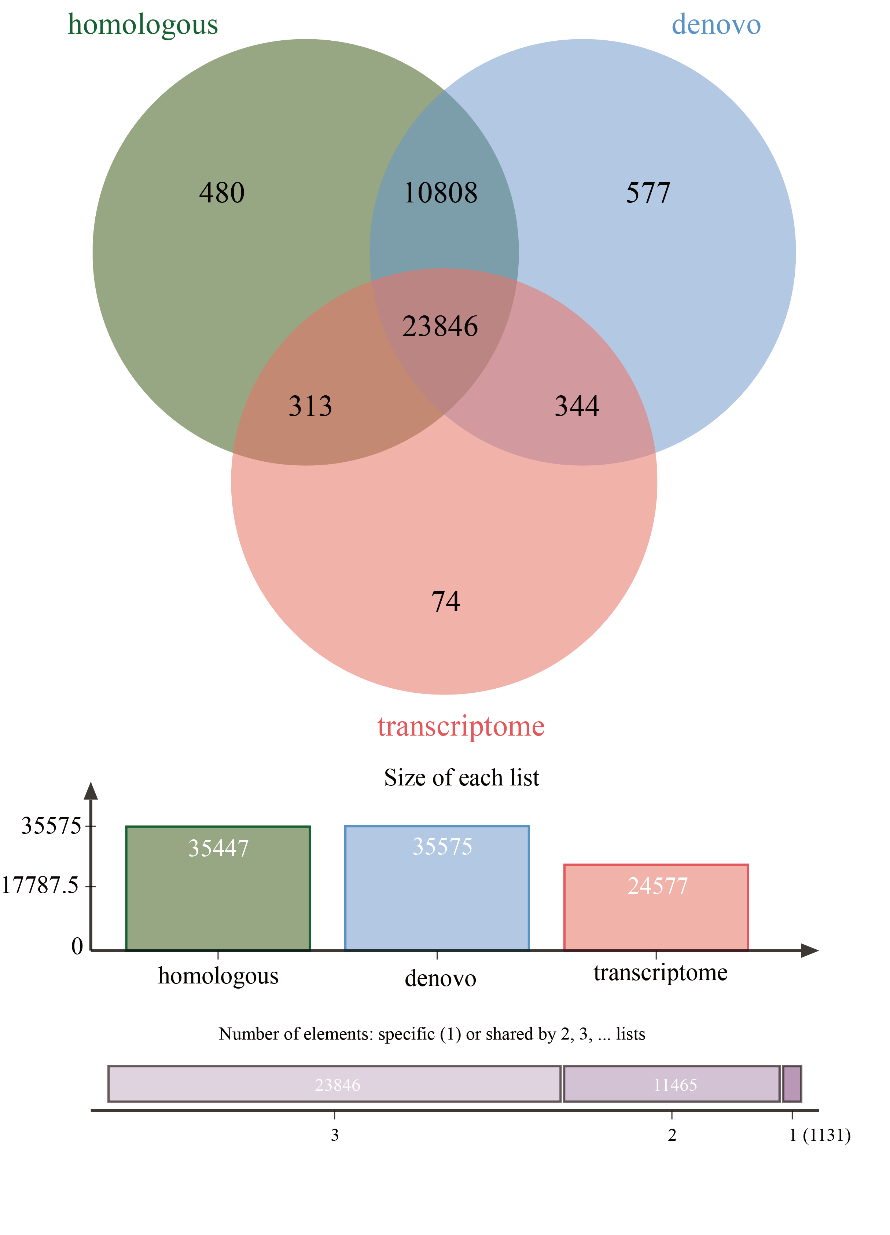


**Supplementary Figure 5.** Protein-coding genes in the *Quercus gilva* genome predicted through a combination of de novo, homology-based and transcriptome-based approaches.


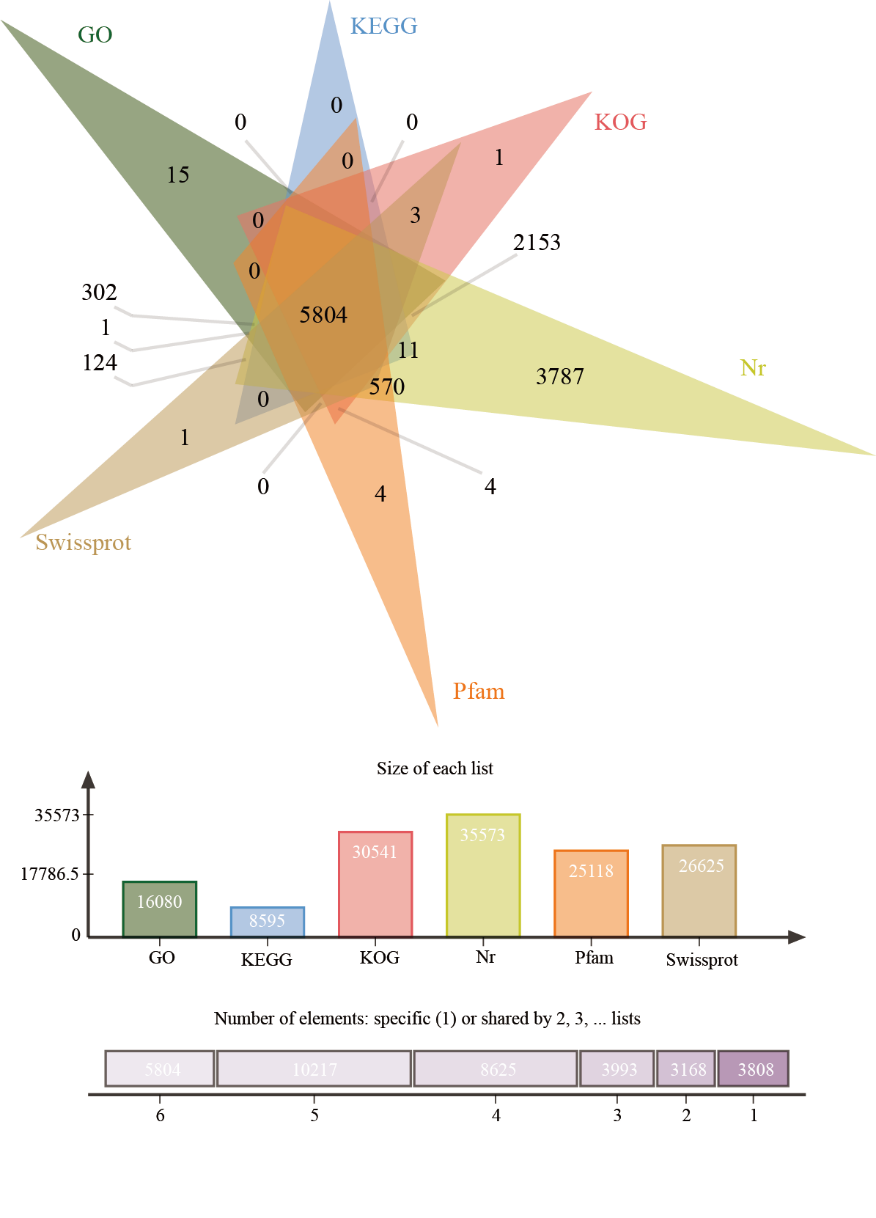


Supplementary Figure 6. Protein-coding genes in the *Quercus gilva* genome functionally annotated in NR (non-redundant), Swiss-prot, KOG (Eukaryotic Orthologous Groups), GO (Gene Ontology), KEGG (Kyoto Encyclopedia of Genes and Genomes) and Pfam databases.


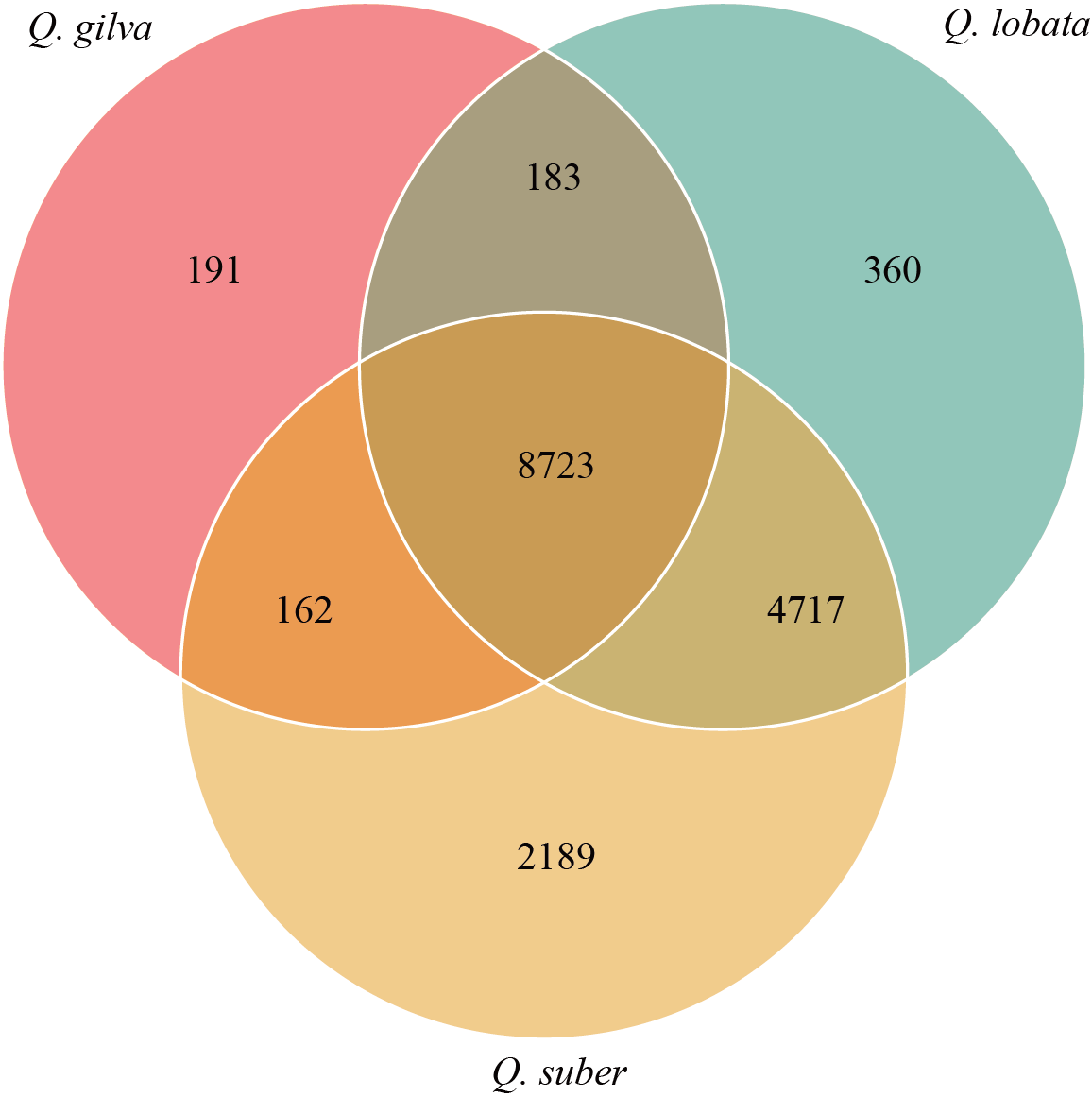


Supplementary Figure 7. Shared and unique gene families in *Quercus gilva*, *Q. lobata* and *Q. suber*.


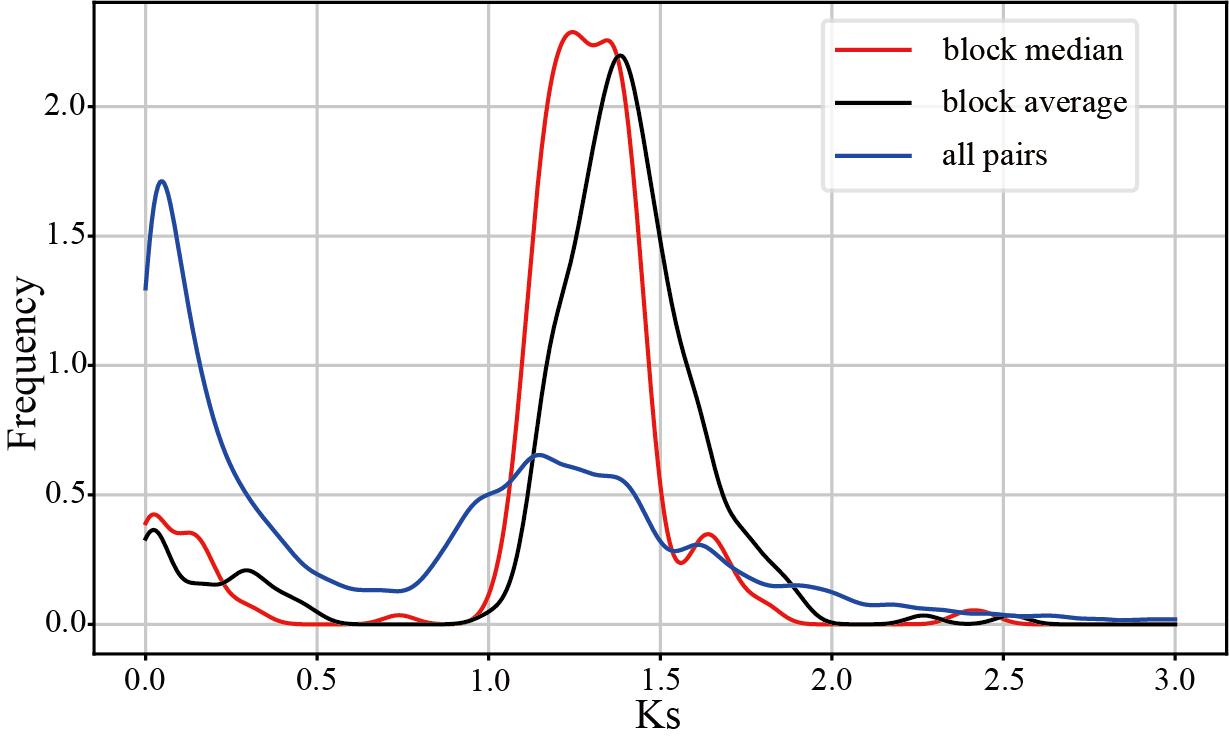


Supplementary Figure 8. Ks (synonymous substitution rate) density distribution for paralogous gene pairs of *Quercus gilva*.


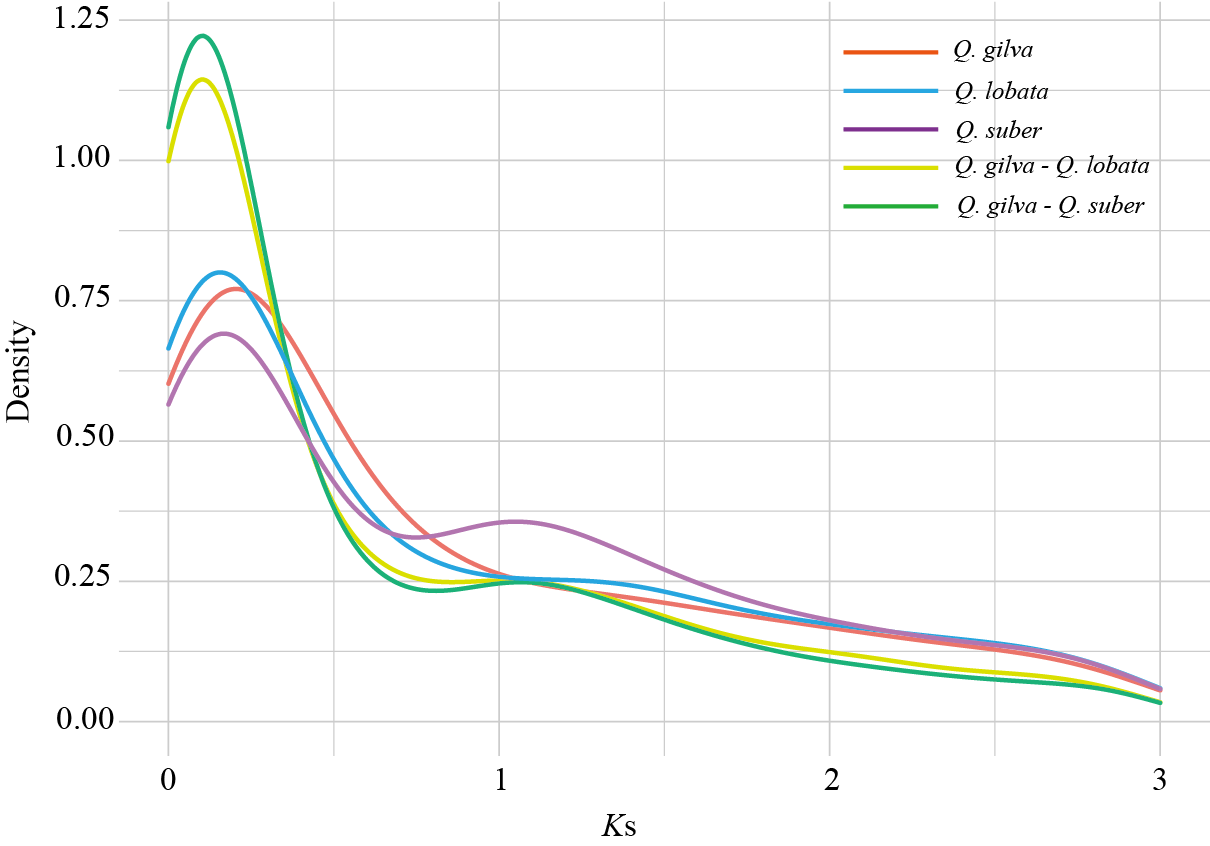


Supplementary Figure 9. Ks (synonymous substitution rate) density distributions for gene pairs of *Quercus gilva*, *Q. lobata*, *Q. suber*, *Q. gilva* versus *Q. lobata*, and *Q. gilva* versus *Q. suber* shown in colored lines as indicated.

## Supplementary Tables

Supplementary Table 1. Summary of genome and transcriptome sequencing information for *Quercus gilva*.

| Read type | Sequencing platform | Tissue | Insert size  (bp) | Clean data  (Gb) | Average subread length (bp) | Sequence coverage  (×)^a^ |
| --- | --- | --- | --- | --- | --- | --- |
| Illumina pair-end reads | Illumina NovaSeq 6000 | Leaves | 350 | 55.66 | 2×150 | 64 |
| PacBio HiFi CCS reads | PacBio Sequel II | Leaves | 20,000 | 30.76 | 15,373 | 36 |
| Hi-C pair-end reads | Illumina NovaSeq 6000 | Leaves | 100-500 | 121.70 | 2×150 | 141 |
| RNA-sequencing reads | Illumina NovaSeq 6000 | Roots, stems, leaves | 350 | 5.74 | 2×150 | - |

^a^ Sequence coverage was calculated using an estimated genome size of 865.75 Mb.

Supplementary Table 2. Genome survey of *Quercus gilva*.

|  | K-mer | K-mer number | K-mer depth | Filtered K-mer number | Genome size (Mb) | Heterozygosity ratio (%) | Repeat (%) |
| --- | --- | --- | --- | --- | --- | --- | --- |
| Value | 19 | 48,949,356,867 | 54.857101 | 47,492,571,457 | 865.75 | 1.16 | 48.17 |

The survey analysis was performed based on Illumina clean data, and a 19 bp length of K-mer was selected. The genome size is estimated using the following formula: Genome size = K-mer Number / K-mer Depth. After filtering error K-mers, the genome size was estimated to be 865.75 Mb.

Supplementary Table 3. Statistics of chromosome-scale genome assembly of *Quercus gilva*.

| Chromosome | Length of contigs (bp) | Length of Chromosome (bp) | Number of Genes |
| --- | --- | --- | --- |
| Chromosome01 | 65,534,972 | 65,536,472 | 2879 |
| Chromosome02 | 101,111,867 | 101,113,867 | 4774 |
| Chromosome03 | 82,144,880 | 82,147,925 | 3107 |
| Chromosome04 | 104,141,225 | 104,151,725 | 3668 |
| Chromosome05 | 100,614,301 | 100,616,801 | 3624 |
| Chromosome06 | 53,748,873 | 53,749,873 | 2659 |
| Chromosome07 | 46,158,203 | 46,159,203 | 2239 |
| Chromosome08 | 70,349,190 | 70,354,190 | 3175 |
| Chromosome09 | 67,951,495 | 67,952,995 | 2677 |
| Chromosome10 | 63,929,837 | 63,930,837 | 2707 |
| Chromosome11 | 63,086,637 | 63,089,637 | 2728 |
| Chromosome12 | 40,259,851 | 40,262,351 | 2205 |
| Total | 859,031,331 | 859,065,876 | 36,442 |
| Genome size | 889,714,170 | 889,842,715 | - |

Supplementary Table 4. Assessment of the *Quercus gilva* genome consistency based on Illumina and PacBio CCS clean reads.

| Statistics | Illumina clean reads | PacBio CSS clean reads |
| --- | --- | --- |
| Mapped reads | 378,923,692 | 2,922,482 |
| Mapped bases (bp) | 53,926,719,419 | 30,107,323,338 |
| Mapping rate (%) | 100.00 | 99.85 |
| Average coverage (×) | 60.60 | 33.83 |
| Coverage rate (%) | 96.22 | 99.87 |

Supplementary Table 5. Classification of repetitive elements in the *Quercus gilva* genome.

| Classification | | | Number of elements | Length of sequence (bp) | Percentage of sequence (%) |
| --- | --- | --- | --- | --- | --- |
| Interspersed repeats (TEs) |  |  | 1,301,159 | 512,260,767 | 57.57 |
|  | SINE |  | 1,171 | 1,798,260 | 0.20 |
|  |  | Other | 1,171 | 1,798,260 | 0.20 |
|  | LINE |  | 57,796 | 34,610,850 | 3.89 |
|  |  | L1/CIN4 | 48,965 | 30,639,498 | 3.44 |
|  |  | RTE/Bov-B | 6,833 | 1,443,944 | 0.16 |
|  |  | Other | 1,998 | 2,527,408 | 0.28 |
|  | LTR |  | 109,621 | 166,161,334 | 18.67 |
|  |  | Copia/Ty1 | 59,143 | 92,483,581 | 10.39 |
|  |  | Gypsy/DIRS1 | 43,591 | 67,347,938 | 7.57 |
|  |  | Other | 186 | 63,944 | 0.01 |
|  |  | Unknown | 6,701 | 6,265,871 | 0.70 |
|  | DNA |  | 40,988 | 21,476,563 | 2.41 |
|  |  | hobo-Activator | 18,088 | 5,412,084 | 0.61 |
|  |  | Tourist/Harbinger | 3,487 | 1,975,542 | 0.22 |
|  |  | Other | 24 | 1,397 | 0.00 |
|  |  | Unknown | 12,507 | 9,686,486 | 1.09 |
|  |  | RC | 6,882 | 4,401,054 | 0.49 |
|  | Unclassified |  | 1,091,583 | 288,213,760 | 32.39 |
| Tandem repeats |  |  | 929,678 | 23,380,270 | 2.63 |

Supplementary Table 6. Statistics of gene structure of the *Quercus gilva* genome.

| Statistics | Value |
| --- | --- |
| Total protein coding genes | 36,442 |
| Average gene length (kb) | 3.7 |
| CDS mean length per gene(kb) | 1.0 |
| Average CDS per gene | 4.4 |
| Average exon length per gene (kb) | 1.1 |
| Average exon per gene | 4.5 |

Supplementary Table 7. Summary of functional annotation of the predicted genes in *Quercus gilva*.

| Type | | Gene number | Percent (%) |
| --- | --- | --- | --- |
| Annotated | | 35,615 | 97.73 |
|  | GO | 16,080 | 44.12 |
|  | NR | 35,573 | 97.62 |
|  | Swiss-prot | 26,625 | 73.06 |
|  | KOG | 30,541 | 83.81 |
|  | Pfam | 25,118 | 68.93 |
|  | KEGG | 8,595 | 23.59 |
| Unannotated | | 827 | 2.27 |
| Total | | 36,442 | 100.00 |

Supplementary Table 8. Clustering statistics of gene families in *Quercus gilva* and nine other plant species.

| Species | Clustered genes | Gene families | Unique gene families | Average genes per family | Single copy genes |
| --- | --- | --- | --- | --- | --- |
| *Arabidopsis thaliana* | 21,714 | 12,110 | 667 | 1.793 | 1,244 |
| *Betula platyphylla* | 22,784 | 12,744 | 309 | 1.788 |  |
| *Castanea dentata* | 22,316 | 13,774 | 33 | 1.620 |  |
| *Fagus sylvatica* | 38,161 | 13,855 | 1,035 | 2.754 |  |
| *Oryza sativa* | 22,651 | 12,032 | 1,203 | 1.883 |  |
| *Populus trichocarpa* | 20,682 | 12,810 | 214 | 1.615 |  |
| *Quercus gilva* | 13,241 | 9,259 | 44 | 1.430 |  |
| *Quercus lobata* | 25,308 | 13,983 | 92 | 1.810 |  |
| *Quercus suber* | 30,517 | 15,791 | 1,877 | 1.933 |  |
| *Ricinus communis* | 17,853 | 12,759 | 125 | 1.399 |  |

Supplementary Table 9. Gene ontology (GO) enrichment analysis of the expanded gene families in *Quercus gilva*.

| GO class | GO ID | GO term | Number of enriched genes | Number of genes in background | *P*-value | *Q*-value |
| --- | --- | --- | --- | --- | --- | --- |
| Biological process | GO:0001101 | response to acid chemical | 9 | 1528 | 0.00000 | 0.00000 |
| Biological process | GO:0006139 | nucleobase-containing compound metabolic process | 6 | 2253 | 0.00299 | 0.00371 |
| Biological process | GO:0006464 | cellular protein modification process | 21 | 2398 | 0.00000 | 0.00000 |
| Biological process | GO:0006468 | protein phosphorylation | 21 | 1230 | 0.00000 | 0.00000 |
| Biological process | GO:0006725 | cellular aromatic compound metabolic process | 6 | 2727 | 0.00823 | 0.00933 |
| Biological process | GO:0006793 | phosphorus metabolic process | 26 | 2010 | 0.00000 | 0.00000 |
| Biological process | GO:0006796 | phosphate-containing compound metabolic process | 26 | 1978 | 0.00000 | 0.00000 |
| Biological process | GO:0006807 | nitrogen compound metabolic process | 28 | 6140 | 0.00000 | 0.00000 |
| Biological process | GO:0006810 | transport | 27 | 2295 | 0.00000 | 0.00000 |
| Biological process | GO:0006811 | ion transport | 6 | 993 | 0.00002 | 0.00005 |
| Biological process | GO:0006812 | cation transport | 6 | 634 | 0.00000 | 0.00000 |
| Biological process | GO:0006950 | response to stress | 17 | 3749 | 0.00000 | 0.00000 |
| Biological process | GO:0006952 | defense response | 16 | 1452 | 0.00000 | 0.00000 |
| Biological process | GO:0006979 | response to oxidative stress | 6 | 528 | 0.00000 | 0.00000 |
| Biological process | GO:0006996 | organelle organization | 5 | 1830 | 0.00452 | 0.00543 |
| Biological process | GO:0007154 | cell communication | 8 | 1998 | 0.00005 | 0.00010 |
| Biological process | GO:0007165 | signal transduction | 8 | 1753 | 0.00002 | 0.00004 |
| Biological process | GO:0007166 | cell surface receptor signaling pathway | 5 | 414 | 0.00000 | 0.00000 |
| Biological process | GO:0007275 | multicellular organism development | 6 | 2597 | 0.00639 | 0.00743 |
| Biological process | GO:0008152 | metabolic process | 37 | 8700 | 0.00000 | 0.00000 |
| Biological process | GO:0009058 | biosynthetic process | 7 | 3015 | 0.00402 | 0.00488 |
| Biological process | GO:0009059 | macromolecule biosynthetic process | 5 | 1337 | 0.00096 | 0.00134 |
| Biological process | GO:0009605 | response to external stimulus | 20 | 1815 | 0.00000 | 0.00000 |
| Biological process | GO:0009607 | response to biotic stimulus | 20 | 1445 | 0.00000 | 0.00000 |
| Biological process | GO:0009617 | response to bacterium | 8 | 715 | 0.00000 | 0.00000 |
| Biological process | GO:0009719 | response to endogenous stimulus | 10 | 2012 | 0.00000 | 0.00000 |
| Biological process | GO:0009725 | response to hormone | 7 | 1905 | 0.00022 | 0.00037 |
| Biological process | GO:0009791 | post-embryonic development | 5 | 1554 | 0.00204 | 0.00259 |
| Biological process | GO:0009889 | regulation of biosynthetic process | 7 | 2174 | 0.00053 | 0.00077 |
| Biological process | GO:0009893 | positive regulation of metabolic process | 8 | 1063 | 0.00000 | 0.00000 |
| Biological process | GO:0009966 | regulation of signal transduction | 8 | 587 | 0.00000 | 0.00000 |
| Biological process | GO:0009987 | cellular process | 40 | 10446 | 0.00000 | 0.00000 |
| Biological process | GO:0010033 | response to organic substance | 13 | 2491 | 0.00000 | 0.00000 |
| Biological process | GO:0010468 | regulation of gene expression | 7 | 2280 | 0.00072 | 0.00102 |
| Biological process | GO:0010556 | regulation of macromolecule biosynthetic process | 7 | 2076 | 0.00039 | 0.00059 |
| Biological process | GO:0010604 | positive regulation of macromolecule metabolic process | 7 | 969 | 0.00000 | 0.00001 |
| Biological process | GO:0010646 | regulation of cell communication | 8 | 615 | 0.00000 | 0.00000 |
| Biological process | GO:0016310 | phosphorylation | 22 | 1442 | 0.00000 | 0.00000 |
| Biological process | GO:0018130 | heterocycle biosynthetic process | 6 | 733 | 0.00000 | 0.00001 |
| Biological process | GO:0019222 | regulation of metabolic process | 8 | 2989 | 0.00100 | 0.00138 |
| Biological process | GO:0019438 | aromatic compound biosynthetic process | 5 | 860 | 0.00009 | 0.00017 |
| Biological process | GO:0019538 | protein metabolic process | 21 | 3544 | 0.00000 | 0.00000 |
| Biological process | GO:0023051 | regulation of signaling | 8 | 612 | 0.00000 | 0.00000 |
| Biological process | GO:0023052 | signaling | 8 | 1783 | 0.00002 | 0.00005 |
| Biological process | GO:0030001 | metal ion transport | 6 | 424 | 0.00000 | 0.00000 |
| Biological process | GO:0031323 | regulation of cellular metabolic process | 8 | 2691 | 0.00048 | 0.00071 |
| Biological process | GO:0031325 | positive regulation of cellular metabolic process | 8 | 993 | 0.00000 | 0.00000 |
| Biological process | GO:0031326 | regulation of cellular biosynthetic process | 7 | 2144 | 0.00048 | 0.00071 |
| Biological process | GO:0034641 | cellular nitrogen compound metabolic process | 7 | 3082 | 0.00458 | 0.00548 |
| Biological process | GO:0034645 | cellular macromolecule biosynthetic process | 5 | 1275 | 0.00075 | 0.00107 |
| Biological process | GO:0034654 | nucleobase-containing compound biosynthetic process | 5 | 487 | 0.00000 | 0.00001 |
| Biological process | GO:0035556 | intracellular signal transduction | 6 | 569 | 0.00000 | 0.00000 |
| Biological process | GO:0036211 | protein modification process | 21 | 2398 | 0.00000 | 0.00000 |
| Biological process | GO:0042221 | response to chemical | 14 | 3530 | 0.00000 | 0.00000 |
| Biological process | GO:0042742 | defense response to bacterium | 8 | 565 | 0.00000 | 0.00000 |
| Biological process | GO:0043170 | macromolecule metabolic process | 26 | 5537 | 0.00000 | 0.00000 |
| Biological process | GO:0043207 | response to external biotic stimulus | 20 | 1422 | 0.00000 | 0.00000 |
| Biological process | GO:0043412 | macromolecule modification | 21 | 2925 | 0.00000 | 0.00000 |
| Biological process | GO:0044237 | cellular metabolic process | 37 | 7519 | 0.00000 | 0.00000 |
| Biological process | GO:0044238 | primary metabolic process | 27 | 6937 | 0.00000 | 0.00000 |
| Biological process | GO:0044249 | cellular biosynthetic process | 7 | 2598 | 0.00164 | 0.00216 |
| Biological process | GO:0044260 | cellular macromolecule metabolic process | 26 | 4248 | 0.00000 | 0.00000 |
| Biological process | GO:0044267 | cellular protein metabolic process | 21 | 3248 | 0.00000 | 0.00000 |
| Biological process | GO:0044271 | cellular nitrogen compound biosynthetic process | 6 | 1274 | 0.00011 | 0.00019 |
| Biological process | GO:0046483 | heterocycle metabolic process | 7 | 2581 | 0.00157 | 0.00208 |
| Biological process | GO:0048518 | positive regulation of biological process | 8 | 1704 | 0.00002 | 0.00003 |
| Biological process | GO:0048519 | negative regulation of biological process | 5 | 1473 | 0.00157 | 0.00208 |
| Biological process | GO:0048522 | positive regulation of cellular process | 8 | 1339 | 0.00000 | 0.00001 |
| Biological process | GO:0048523 | negative regulation of cellular process | 5 | 1095 | 0.00034 | 0.00053 |
| Biological process | GO:0048583 | regulation of response to stimulus | 9 | 1162 | 0.00000 | 0.00000 |
| Biological process | GO:0050789 | regulation of biological process | 15 | 5363 | 0.00001 | 0.00003 |
| Biological process | GO:0050794 | regulation of cellular process | 14 | 4766 | 0.00001 | 0.00003 |
| Biological process | GO:0050896 | response to stimulus | 33 | 6417 | 0.00000 | 0.00000 |
| Biological process | GO:0051171 | regulation of nitrogen compound metabolic process | 7 | 2464 | 0.00118 | 0.00161 |
| Biological process | GO:0051173 | positive regulation of nitrogen compound metabolic process | 7 | 919 | 0.00000 | 0.00000 |
| Biological process | GO:0051179 | localization | 27 | 2515 | 0.00000 | 0.00000 |
| Biological process | GO:0051234 | establishment of localization | 27 | 2327 | 0.00000 | 0.00000 |
| Biological process | GO:0051704 | multi-organism process | 20 | 1871 | 0.00000 | 0.00000 |
| Biological process | GO:0051707 | response to other organism | 20 | 1407 | 0.00000 | 0.00000 |
| Biological process | GO:0051716 | cellular response to stimulus | 9 | 3105 | 0.00032 | 0.00049 |
| Biological process | GO:0055085 | transmembrane transport | 24 | 1265 | 0.00000 | 0.00000 |
| Biological process | GO:0060255 | regulation of macromolecule metabolic process | 7 | 2685 | 0.00200 | 0.00255 |
| Biological process | GO:0065007 | biological regulation | 15 | 6160 | 0.00007 | 0.00014 |
| Biological process | GO:0070887 | cellular response to chemical stimulus | 6 | 1473 | 0.00026 | 0.00042 |
| Biological process | GO:0071310 | cellular response to organic substance | 6 | 1076 | 0.00004 | 0.00007 |
| Biological process | GO:0071704 | organic substance metabolic process | 28 | 7671 | 0.00000 | 0.00000 |
| Biological process | GO:0080090 | regulation of primary metabolic process | 8 | 2531 | 0.00031 | 0.00048 |
| Biological process | GO:0090304 | nucleic acid metabolic process | 5 | 1929 | 0.00580 | 0.00681 |
| Biological process | GO:0098542 | defense response to other organism | 13 | 1016 | 0.00000 | 0.00000 |
| Biological process | GO:1901360 | organic cyclic compound metabolic process | 7 | 2941 | 0.00347 | 0.00426 |
| Biological process | GO:1901362 | organic cyclic compound biosynthetic process | 6 | 1010 | 0.00003 | 0.00005 |
| Biological process | GO:1901564 | organonitrogen compound metabolic process | 23 | 4442 | 0.00000 | 0.00000 |
| Biological process | GO:1901576 | organic substance biosynthetic process | 7 | 2736 | 0.00225 | 0.00280 |
| Biological process | GO:1901700 | response to oxygen-containing compound | 13 | 2168 | 0.00000 | 0.00000 |
| Biological process | GO:1901701 | cellular response to oxygen-containing compound | 6 | 781 | 0.00000 | 0.00001 |
| Biological process | GO:2000112 | regulation of cellular macromolecule biosynthetic process | 7 | 2037 | 0.00035 | 0.00053 |
| Cellular component | GO:0000325 | plant-type vacuole | 16 | 298 | 0.00000 | 0.00000 |
| Cellular component | GO:0005576 | extracellular region | 8 | 639 | 0.00000 | 0.00000 |
| Cellular component | GO:0005622 | intracellular | 37 | 11235 | 0.00000 | 0.00000 |
| Cellular component | GO:0005623 | cell | 59 | 13525 | 0.00000 | 0.00000 |
| Cellular component | GO:0005634 | nucleus | 5 | 3866 | 0.00273 | 0.00338 |
| Cellular component | GO:0005737 | cytoplasm | 32 | 8656 | 0.00000 | 0.00000 |
| Cellular component | GO:0005739 | mitochondrion | 6 | 1088 | 0.00000 | 0.00000 |
| Cellular component | GO:0005773 | vacuole | 22 | 1251 | 0.00000 | 0.00000 |
| Cellular component | GO:0005774 | vacuolar membrane | 16 | 591 | 0.00000 | 0.00000 |
| Cellular component | GO:0005829 | cytosol | 5 | 2190 | 0.00014 | 0.00025 |
| Cellular component | GO:0005856 | cytoskeleton | 8 | 440 | 0.00000 | 0.00000 |
| Cellular component | GO:0005886 | plasma membrane | 29 | 3649 | 0.00000 | 0.00000 |
| Cellular component | GO:0005911 | cell-cell junction | 15 | 1139 | 0.00000 | 0.00000 |
| Cellular component | GO:0009506 | plasmodesma | 15 | 1114 | 0.00000 | 0.00000 |
| Cellular component | GO:0016020 | membrane | 45 | 5913 | 0.00000 | 0.00000 |
| Cellular component | GO:0016021 | integral component of membrane | 5 | 711 | 0.00000 | 0.00000 |
| Cellular component | GO:0030054 | cell junction | 15 | 1152 | 0.00000 | 0.00000 |
| Cellular component | GO:0031090 | organelle membrane | 16 | 1260 | 0.00000 | 0.00000 |
| Cellular component | GO:0031224 | intrinsic component of membrane | 5 | 933 | 0.00000 | 0.00000 |
| Cellular component | GO:0032991 | protein-containing complex | 9 | 2713 | 0.00000 | 0.00000 |
| Cellular component | GO:0043226 | organelle | 36 | 9865 | 0.00000 | 0.00000 |
| Cellular component | GO:0043227 | membrane-bounded organelle | 33 | 9507 | 0.00000 | 0.00000 |
| Cellular component | GO:0043228 | non-membrane-bounded organelle | 12 | 1956 | 0.00000 | 0.00000 |
| Cellular component | GO:0043229 | intracellular organelle | 36 | 9827 | 0.00000 | 0.00000 |
| Cellular component | GO:0043231 | intracellular membrane-bounded organelle | 33 | 9339 | 0.00000 | 0.00000 |
| Cellular component | GO:0043232 | intracellular non-membrane-bounded organelle | 12 | 1956 | 0.00000 | 0.00000 |
| Cellular component | GO:0044422 | organelle part | 26 | 4715 | 0.00000 | 0.00000 |
| Cellular component | GO:0044424 | intracellular part | 37 | 11201 | 0.00000 | 0.00000 |
| Cellular component | GO:0044425 | membrane part | 5 | 1694 | 0.00004 | 0.00007 |
| Cellular component | GO:0044428 | nuclear part | 5 | 1558 | 0.00002 | 0.00005 |
| Cellular component | GO:0044430 | cytoskeletal part | 5 | 354 | 0.00000 | 0.00000 |
| Cellular component | GO:0044437 | vacuolar part | 16 | 598 | 0.00000 | 0.00000 |
| Cellular component | GO:0044444 | cytoplasmic part | 32 | 7488 | 0.00000 | 0.00000 |
| Cellular component | GO:0044446 | intracellular organelle part | 26 | 4676 | 0.00000 | 0.00000 |
| Cellular component | GO:0044464 | cell part | 59 | 13525 | 0.00000 | 0.00000 |
| Cellular component | GO:0048046 | apoplast | 8 | 348 | 0.00000 | 0.00000 |
| Cellular component | GO:0055044 | symplast | 15 | 1114 | 0.00000 | 0.00000 |
| Cellular component | GO:0071944 | cell periphery | 31 | 4272 | 0.00000 | 0.00000 |
| Cellular component | GO:0098588 | bounding membrane of organelle | 16 | 917 | 0.00000 | 0.00000 |
| Molecular function | GO:0003824 | catalytic activity | 52 | 7557 | 0.00000 | 0.00000 |
| Molecular function | GO:0004672 | protein kinase activity | 21 | 1210 | 0.00000 | 0.00000 |
| Molecular function | GO:0004674 | protein serine/threonine kinase activity | 20 | 1029 | 0.00000 | 0.00000 |
| Molecular function | GO:0005215 | transporter activity | 24 | 1279 | 0.00000 | 0.00000 |
| Molecular function | GO:0005488 | binding | 10 | 5426 | 0.00134 | 0.00181 |
| Molecular function | GO:0005515 | protein binding | 10 | 2221 | 0.00000 | 0.00000 |
| Molecular function | GO:0005516 | calmodulin binding | 5 | 290 | 0.00000 | 0.00000 |
| Molecular function | GO:0010290 | chlorophyll catabolite transmembrane transporter activity | 8 | 18 | 0.00000 | 0.00000 |
| Molecular function | GO:0015399 | primary active transmembrane transporter activity | 21 | 260 | 0.00000 | 0.00000 |
| Molecular function | GO:0015431 | glutathione S-conjugate-exporting ATPase activity | 8 | 18 | 0.00000 | 0.00000 |
| Molecular function | GO:0016301 | kinase activity | 22 | 1378 | 0.00000 | 0.00000 |
| Molecular function | GO:0016462 | pyrophosphatase activity | 25 | 796 | 0.00000 | 0.00000 |
| Molecular function | GO:0016740 | transferase activity | 23 | 3129 | 0.00000 | 0.00000 |
| Molecular function | GO:0016772 | transferase activity, transferring phosphorus-containing groups | 22 | 1509 | 0.00000 | 0.00000 |
| Molecular function | GO:0016773 | phosphotransferase activity, alcohol group as acceptor | 21 | 1330 | 0.00000 | 0.00000 |
| Molecular function | GO:0016787 | hydrolase activity | 29 | 2717 | 0.00000 | 0.00000 |
| Molecular function | GO:0016817 | hydrolase activity, acting on acid anhydrides | 25 | 807 | 0.00000 | 0.00000 |
| Molecular function | GO:0016818 | hydrolase activity, acting on acid anhydrides, in phosphorus-containing anhydrides | 25 | 801 | 0.00000 | 0.00000 |
| Molecular function | GO:0016887 | ATPase activity | 25 | 648 | 0.00000 | 0.00000 |
| Molecular function | GO:0017111 | nucleoside-triphosphatase activity | 25 | 749 | 0.00000 | 0.00000 |
| Molecular function | GO:0022804 | active transmembrane transporter activity | 21 | 473 | 0.00000 | 0.00000 |
| Molecular function | GO:0022857 | transmembrane transporter activity | 24 | 1170 | 0.00000 | 0.00000 |
| Molecular function | GO:0042626 | ATPase activity, coupled to transmembrane movement of substances | 21 | 246 | 0.00000 | 0.00000 |
| Molecular function | GO:0097159 | organic cyclic compound binding | 7 | 2936 | 0.00102 | 0.00141 |
| Molecular function | GO:0140096 | catalytic activity, acting on a protein | 21 | 2381 | 0.00000 | 0.00000 |
| Molecular function | GO:1901363 | heterocyclic compound binding | 7 | 2914 | 0.00097 | 0.00135 |

*Q*-value less than 0.01 is considered to be very significant.

Only the GO terms of which the number of enriched genes≥5 are listed in the table.

Supplementary Table 10. Gene ontology (GO) enrichment analysis of the contracted gene families in *Quercus gilva*.

| GO class | GO ID | GO term | Number of enriched genes | Number of genes in background | *P*-value | *Q*-value |
| --- | --- | --- | --- | --- | --- | --- |
| Biological process | GO:0001101 | response to acid chemical | 8 | 1528 | 0.00001 | 0.00011 |
| Biological process | GO:0002376 | immune system process | 21 | 767 | 0.00000 | 0.00000 |
| Biological process | GO:0006464 | cellular protein modification process | 17 | 2398 | 0.00000 | 0.00000 |
| Biological process | GO:0006468 | protein phosphorylation | 17 | 1230 | 0.00000 | 0.00000 |
| Biological process | GO:0006793 | phosphorus metabolic process | 17 | 2010 | 0.00000 | 0.00000 |
| Biological process | GO:0006796 | phosphate-containing compound metabolic process | 17 | 1978 | 0.00000 | 0.00000 |
| Biological process | GO:0006807 | nitrogen compound metabolic process | 18 | 6140 | 0.00001 | 0.00005 |
| Biological process | GO:0006950 | response to stress | 24 | 3749 | 0.00000 | 0.00000 |
| Biological process | GO:0006952 | defense response | 22 | 1452 | 0.00000 | 0.00000 |
| Biological process | GO:0006955 | immune response | 21 | 678 | 0.00000 | 0.00000 |
| Biological process | GO:0007154 | cell communication | 27 | 1998 | 0.00000 | 0.00000 |
| Biological process | GO:0007165 | signal transduction | 27 | 1753 | 0.00000 | 0.00000 |
| Biological process | GO:0007166 | cell surface receptor signaling pathway | 12 | 414 | 0.00000 | 0.00000 |
| Biological process | GO:0007167 | enzyme linked receptor protein signaling pathway | 9 | 277 | 0.00000 | 0.00000 |
| Biological process | GO:0007178 | transmembrane receptor protein serine/threonine kinase signaling pathway | 9 | 272 | 0.00000 | 0.00000 |
| Biological process | GO:0008152 | metabolic process | 20 | 8700 | 0.00007 | 0.00037 |
| Biological process | GO:0008219 | cell death | 15 | 365 | 0.00000 | 0.00000 |
| Biological process | GO:0009626 | plant-type hypersensitive response | 14 | 200 | 0.00000 | 0.00000 |
| Biological process | GO:0009719 | response to endogenous stimulus | 8 | 2012 | 0.00012 | 0.00045 |
| Biological process | GO:0009725 | response to hormone | 8 | 1905 | 0.00008 | 0.00041 |
| Biological process | GO:0009751 | response to salicylic acid | 5 | 293 | 0.00000 | 0.00000 |
| Biological process | GO:0009987 | cellular process | 37 | 10446 | 0.00000 | 0.00000 |
| Biological process | GO:0010033 | response to organic substance | 9 | 2491 | 0.00012 | 0.00045 |
| Biological process | GO:0012501 | programmed cell death | 15 | 339 | 0.00000 | 0.00000 |
| Biological process | GO:0014070 | response to organic cyclic compound | 6 | 642 | 0.00000 | 0.00002 |
| Biological process | GO:0016310 | phosphorylation | 17 | 1442 | 0.00000 | 0.00000 |
| Biological process | GO:0019538 | protein metabolic process | 18 | 3544 | 0.00000 | 0.00000 |
| Biological process | GO:0023052 | signaling | 27 | 1783 | 0.00000 | 0.00000 |
| Biological process | GO:0032501 | multicellular organismal process | 7 | 2886 | 0.00554 | 0.00933 |
| Biological process | GO:0033554 | cellular response to stress | 16 | 1291 | 0.00000 | 0.00000 |
| Biological process | GO:0034050 | host programmed cell death induced by symbiont | 14 | 200 | 0.00000 | 0.00000 |
| Biological process | GO:0036211 | protein modification process | 17 | 2398 | 0.00000 | 0.00000 |
| Biological process | GO:0042221 | response to chemical | 10 | 3530 | 0.00049 | 0.00126 |
| Biological process | GO:0043170 | macromolecule metabolic process | 18 | 5537 | 0.00000 | 0.00001 |
| Biological process | GO:0043412 | macromolecule modification | 17 | 2925 | 0.00000 | 0.00000 |
| Biological process | GO:0044237 | cellular metabolic process | 19 | 7519 | 0.00003 | 0.00018 |
| Biological process | GO:0044238 | primary metabolic process | 18 | 6937 | 0.00003 | 0.00019 |
| Biological process | GO:0044260 | cellular macromolecule metabolic process | 18 | 4248 | 0.00000 | 0.00000 |
| Biological process | GO:0044267 | cellular protein metabolic process | 18 | 3248 | 0.00000 | 0.00000 |
| Biological process | GO:0045087 | innate immune response | 21 | 649 | 0.00000 | 0.00000 |
| Biological process | GO:0046677 | response to antibiotic | 5 | 435 | 0.00000 | 0.00003 |
| Biological process | GO:0050789 | regulation of biological process | 30 | 5363 | 0.00000 | 0.00000 |
| Biological process | GO:0050794 | regulation of cellular process | 30 | 4766 | 0.00000 | 0.00000 |
| Biological process | GO:0050896 | response to stimulus | 36 | 6417 | 0.00000 | 0.00000 |
| Biological process | GO:0051716 | cellular response to stimulus | 29 | 3105 | 0.00000 | 0.00000 |
| Biological process | GO:0065007 | biological regulation | 31 | 6160 | 0.00000 | 0.00000 |
| Biological process | GO:0071704 | organic substance metabolic process | 18 | 7671 | 0.00011 | 0.00045 |
| Biological process | GO:1901564 | organonitrogen compound metabolic process | 18 | 4442 | 0.00000 | 0.00000 |
| Biological process | GO:1901700 | response to oxygen-containing compound | 9 | 2168 | 0.00004 | 0.00023 |
| Cellular component | GO:0005622 | intracellular | 14 | 11235 | 0.00000 | 0.00004 |
| Cellular component | GO:0005623 | cell | 37 | 13525 | 0.00000 | 0.00000 |
| Cellular component | GO:0005737 | cytoplasm | 13 | 8656 | 0.00000 | 0.00001 |
| Cellular component | GO:0005773 | vacuole | 9 | 1251 | 0.00000 | 0.00000 |
| Cellular component | GO:0005886 | plasma membrane | 35 | 3649 | 0.00000 | 0.00000 |
| Cellular component | GO:0005911 | cell-cell junction | 10 | 1139 | 0.00000 | 0.00000 |
| Cellular component | GO:0009506 | plasmodesma | 9 | 1114 | 0.00000 | 0.00000 |
| Cellular component | GO:0016020 | membrane | 36 | 5913 | 0.00000 | 0.00000 |
| Cellular component | GO:0019897 | extrinsic component of plasma membrane | 12 | 105 | 0.00000 | 0.00000 |
| Cellular component | GO:0019898 | extrinsic component of membrane | 12 | 154 | 0.00000 | 0.00000 |
| Cellular component | GO:0030054 | cell junction | 10 | 1152 | 0.00000 | 0.00000 |
| Cellular component | GO:0043226 | organelle | 14 | 9865 | 0.00000 | 0.00001 |
| Cellular component | GO:0043227 | membrane-bounded organelle | 12 | 9507 | 0.00002 | 0.00013 |
| Cellular component | GO:0043229 | intracellular organelle | 14 | 9827 | 0.00000 | 0.00001 |
| Cellular component | GO:0043231 | intracellular membrane-bounded organelle | 12 | 9339 | 0.00001 | 0.00011 |
| Cellular component | GO:0044424 | intracellular part | 14 | 11201 | 0.00000 | 0.00004 |
| Cellular component | GO:0044425 | membrane part | 14 | 1694 | 0.00000 | 0.00000 |
| Cellular component | GO:0044444 | cytoplasmic part | 13 | 7488 | 0.00000 | 0.00000 |
| Cellular component | GO:0044459 | plasma membrane part | 13 | 709 | 0.00000 | 0.00000 |
| Cellular component | GO:0044464 | cell part | 37 | 13525 | 0.00000 | 0.00000 |
| Cellular component | GO:0055044 | symplast | 9 | 1114 | 0.00000 | 0.00000 |
| Cellular component | GO:0071944 | cell periphery | 35 | 4272 | 0.00000 | 0.00000 |
| Molecular function | GO:0000166 | nucleotide binding | 13 | 520 | 0.00000 | 0.00000 |
| Molecular function | GO:0003674 | molecular function | 34 | 12425 | 0.00000 | 0.00000 |
| Molecular function | GO:0003824 | catalytic activity | 20 | 7557 | 0.00000 | 0.00000 |
| Molecular function | GO:0004672 | protein kinase activity | 17 | 1210 | 0.00000 | 0.00000 |
| Molecular function | GO:0004674 | protein serine/threonine kinase activity | 17 | 1029 | 0.00000 | 0.00000 |
| Molecular function | GO:0004675 | transmembrane receptor protein serine/threonine kinase activity | 9 | 271 | 0.00000 | 0.00000 |
| Molecular function | GO:0004888 | transmembrane signaling receptor activity | 9 | 368 | 0.00000 | 0.00000 |
| Molecular function | GO:0005488 | binding | 27 | 5426 | 0.00000 | 0.00000 |
| Molecular function | GO:0005515 | protein binding | 14 | 2221 | 0.00000 | 0.00000 |
| Molecular function | GO:0016301 | kinase activity | 17 | 1378 | 0.00000 | 0.00000 |
| Molecular function | GO:0016740 | transferase activity | 17 | 3129 | 0.00000 | 0.00000 |
| Molecular function | GO:0016772 | transferase activity, transferring phosphorus-containing groups | 17 | 1509 | 0.00000 | 0.00000 |
| Molecular function | GO:0016773 | phosphotransferase activity, alcohol group as acceptor | 17 | 1330 | 0.00000 | 0.00000 |
| Molecular function | GO:0019199 | transmembrane receptor protein kinase activity | 9 | 278 | 0.00000 | 0.00000 |
| Molecular function | GO:0019899 | enzyme binding | 9 | 566 | 0.00000 | 0.00000 |
| Molecular function | GO:0031625 | ubiquitin protein ligase binding | 8 | 184 | 0.00000 | 0.00000 |
| Molecular function | GO:0036094 | small molecule binding | 13 | 638 | 0.00000 | 0.00000 |
| Molecular function | GO:0038023 | signaling receptor activity | 9 | 413 | 0.00000 | 0.00000 |
| Molecular function | GO:0044389 | ubiquitin-like protein ligase binding | 8 | 185 | 0.00000 | 0.00000 |
| Molecular function | GO:0060089 | molecular transducer activity | 9 | 451 | 0.00000 | 0.00000 |
| Molecular function | GO:0097159 | organic cyclic compound binding | 14 | 2936 | 0.00000 | 0.00000 |
| Molecular function | GO:0140096 | catalytic activity, acting on a protein | 17 | 2381 | 0.00000 | 0.00000 |
| Molecular function | GO:1901265 | nucleoside phosphate binding | 13 | 520 | 0.00000 | 0.00000 |
| Molecular function | GO:1901363 | heterocyclic compound binding | 14 | 2914 | 0.00000 | 0.00000 |

*Q*-value less than 0.01 is considered to be very significant.

Only the GO terms of which the number of enriched genes≥5 are listed in the table.

Supplementary Table 11. KEGG (Kyoto Encyclopedia of Genes and Genomes) enrichment analysis of the expanded gene family in *Quercus gilva*.

| Pathway hierarchy1 | Pathway hierarchy2 | KEGG pathway | Pathway ID | Number of enriched genes | Number of genes in background | *P*-value | *Q*-value |
| --- | --- | --- | --- | --- | --- | --- | --- |
| Brite Hierarchies | Protein families: signaling and cellular processes | Transporters | ko02000 | 8 | 628 | 0.00000 | 0.00000 |
| Environmental Information Processing | Membrane transport | ABC transporters | ko02010 | 8 | 59 | 0.00000 | 0.00000 |

*Q*-value less than 0.01 is considered to be very significant.

Supplementary Table 12. KEGG (Kyoto Encyclopedia of Genes and Genomes) enrichment analysis of the contracted gene family in *Quercus gilva*.

| Pathway hierarchy1 | Pathway hierarchy2 | KEGG pathway | Pathway ID | Number of enriched genes | Number of genes in background | *P*-value | *Q*-value |
| --- | --- | --- | --- | --- | --- | --- | --- |
| Metabolism | Amino acid metabolism | Tryptophan metabolism | ko00380 | 2 | 59 | 0.00007 | 0.00025 |
| Metabolism | Metabolism of other amino acids | Glutathione metabolism | ko00480 | 1 | 63 | 0.00315 | 0.00427 |
| Metabolism | Metabolism of terpenoids and polyketides | Monoterpenoid biosynthesis | ko00902 | 2 | 5 | 0.00000 | 0.00000 |
| Metabolism | Biosynthesis of other secondary metabolites | Phenylpropanoid biosynthesis | ko00940 | 2 | 142 | 0.00087 | 0.00213 |
| Metabolism | Xenobiotics biodegradation and metabolism | Metabolism of xenobiotics by cytochrome P450 | ko00980 | 1 | 34 | 0.00093 | 0.00213 |
| Metabolism | Xenobiotics biodegradation and metabolism | Drug metabolism - cytochrome P450 | ko00982 | 1 | 40 | 0.00128 | 0.00268 |
| Metabolism | Xenobiotics biodegradation and metabolism | Drug metabolism - other enzymes | ko00983 | 1 | 50 | 0.00200 | 0.00328 |
| Human Diseases | Drug resistance: antineoplastic | Platinum drug resistance | ko01524 | 1 | 57 | 0.00259 | 0.00397 |
| Environmental Information Processing | Membrane transport | ABC transporters | ko02010 | 1 | 59 | 0.00277 | 0.00398 |
| Organismal Systems | Aging | Longevity regulating pathway - worm | ko04212 | 1 | 73 | 0.00421 | 0.00509 |
| Organismal Systems | Environmental adaptation | Plant-pathogen interaction | ko04626 | 9 | 214 | 0.00000 | 0.00000 |
| Human Diseases | Cancer: overview | Chemical carcinogenesis - DNA adducts | ko05204 | 1 | 25 | 0.00050 | 0.00144 |
| Human Diseases | Cancer: overview | Chemical carcinogenesis - receptor activation | ko05207 | 1 | 47 | 0.00177 | 0.00326 |
| Human Diseases | Cancer: specific types | Hepatocellular carcinoma | ko05225 | 1 | 48 | 0.00184 | 0.00326 |
| Human Diseases | Cardiovascular disease | Fluid shear stress and atherosclerosis | ko05418 | 1 | 71 | 0.00399 | 0.00509 |

*Q*-value less than 0.01 is considered to be very significant.
